# Using genetic algorithms to systematically improve the synthesis conditions of Al-PMOF

**DOI:** 10.1038/s42004-022-00785-2

**Published:** 2022-12-10

**Authors:** Nency P. Domingues, Seyed Mohamad Moosavi, Leopold Talirz, Kevin Maik Jablonka, Christopher P. Ireland, Fatmah Mish Ebrahim, Berend Smit

**Affiliations:** 1 Laboratory of Molecular Simulation (LSMO), Institut des Sciences et Ingénierie Chimiques, École Polytechnique Fédérale de Lausanne (EPFL), Sion, Valais Switzerland; 2 Department of Mathematics and Computer Science, Freie Universität Berlin, Berlin, Germany; 3 Theory and Simulation of Materials (THEOS), School of Engineering (STI), École Polytechnique Fédérale de Lausanne (EPFL), Lausanne, Vaud Switzerland; 4 Cavendish Laboratory, School of Physical Sciences, University of Cambridge, Cambridge, UK

**Keywords:** Carbon capture and storage, Automation, Metal-organic frameworks, Microwave chemistry

## Abstract

The synthesis of metal-organic frameworks (MOFs) is often complex and the desired structure is not always obtained. In this work, we report a methodology that uses a joint machine learning and experimental approach to optimize the synthesis conditions of Al-PMOF (Al_2_(OH)_2_TCPP) [H_2_TCPP = meso-tetra(4-carboxyphenyl)porphine], a promising material for carbon capture applications. Al-PMOF was previously synthesized using a hydrothermal reaction, which gave a low throughput yield due to its relatively long reaction time (16 hours). Here, we use a genetic algorithm to carry out a systematic search for the optimal synthesis conditions and a microwave-based high-throughput robotic platform for the syntheses. We show that, in just two generations, we could obtain excellent crystallinity and yield close to 80% in a much shorter reaction time (50 minutes). Moreover, by analyzing the failed and partially successful experiments, we could identify the most important experimental variables that determine the crystallinity and yield.

## Abstract (web summary)

Metal-organic frameworks with desirable properties can be designed through careful choice of linker and node combinations, but achieving the synthesis of a desired MOF is complex and dependent on many experimental variables. Here, a genetic algorithm combined with experimental feedback and confirmation is used to obtain the optimal microwave-assisted synthesis conditions for a porphyrin-based aluminium MOF (Al-PMOF), achieving excellent crystallinity and a close to 80% yield in only the 2^nd^ generation.

## Introduction

For the last two decades, metal-organic frameworks (MOFs) have been an extensive object of study^1–3^ thanks to their high porosity^4–7^ and extensive spectrum of applications, including gas storage and separation, sensing, catalysis, and drug delivery^8–17^. MOF synthesis consists of the self-assembly of organic ligands and metal components into a periodic network^18^. Several methods have been developed for such purposes including solvothermal, electrochemical, mechanochemical, microwave, and ultrasound-based methodologies^8,16,19–21^. In all these procedures, the synthesis parameters play a major role in determining the crystal structure that forms, as different conditions might stabilize different (meta)stable (intermediate) states^22^.

There are a considerable number of parameters that can influence the reaction and its outcome (i.e., solvents, pH, reagents concentration, reaction time, temperature, pressure, etc.)^23,24^, and the optimization of these conditions for new or established MOFs is often laborious, expensive and time-consuming^25,26^. While conventionally, the optimization of these parameters rests on the chemical intuition of individuals, novel approaches are needed to tackle the extensive diversity in the chemistry of MOFs^27^. Therefore, data-driven approaches have been developed to accelerate such optimization processes^28–37^. Moosavi et al.^28^ combined a genetic algorithm (GA) with machine learning (ML) to optimize the synthesis of MOFs. They illustrated their approach with the synthesis of HKUST-1^38^ using a microwave-based robotic platform, to find the synthesis conditions of HKUST-1 that yielded high-quality crystals with the highest surface area reported up to date^28^. This approach not only aims to find the optimal reaction conditions, but also to learn the most important experimental variables from analyzing both successful, partially successful, and failed experiments.

**Fig. 1:**
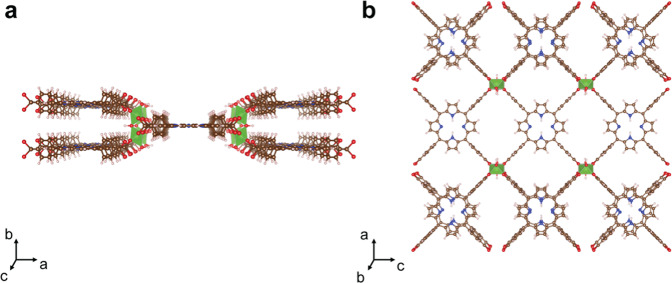
Ball-and-stick representation of Al-PMOF. **a** View along the *c*-axis. **b** View along the *b*-axis. Color code: C (brown), O (red), N (blue), H (light pink), Al (green).

In this work, we applied the Synthetic Conditions Finder (SyCoFinder)^39^, which is the web application based on the methodology developed by Moosavi et al.^28^, to find the optimal synthesis conditions of Al-PMOF (Al_2_(OH)_2_TCPP) [H_2_TCPP = meso-tetra(4-carboxyphenyl)porphine], a porphyrin-based MOF first synthesized by Fateeva et al.^40^. Publications of porphyrin-based MOFs have been exponentially increasing in the past 20 years^41^ as this organic ligand has interesting characteristics and versatile functions, which makes it suitable for a wide range of applications. Thanks to their high visible-light absorption and energy transfer properties, porphyrins are very often used in solar cells, fluorescence imaging, and molecular probe applications^42^. The structure of Al-PMOF relies on one-dimensional chains of Al(III) running along the *b*-axis connected by the TCPP units through the carboxylate groups (Fig. 1a, b). The high surface area of this MOF and the stacks of the porphyrin ligand along the *b*-axis make this structure suitable for CO_2_ capture in a wet environment, typical of flue gas from a coal-fired power station^43^.

Unlike HKUST-1, our knowledge of alternative synthesis conditions of Al-PMOF is limited. Some reports mention its synthesis using different reaction temperatures and aluminum precursors in a DMF:H_2_O = 1:3 [v/v] solvent mixture^44,45^. However, the yield of these reactions is not reported. Moreover, the original hydrothermal synthesis gives a relatively low yield (ca. 40%) with a reaction time of 16 h^40^, and these factors present a bottleneck to scale-up the synthesis for CO_2_ capture from wet flue gas. It is therefore important to investigate whether the yield and time of the reaction can be further optimized. In addition, it will give us some insights into whether the approach developed by Moosavi et al. can be extended to other MOF systems.

## Results

### Experimental variables

The reported Al-PMOF synthesis is in pure water at a relatively high temperature (i.e., 180 °C)^40^. We have carried out some attempts to synthesize Al-PMOF at a lower temperature or in pure dimethylformamide (DMF), which easily dissolves the ligand, but at these conditions, we do not produce the MOF. If we repeat the synthesis in pure water, we obtained variable yields (40–90%) (see Supplementary Note 1 for detailed yields obtained). It is therefore interesting to systematically explore the synthesis conditions. For this purpose, we used our high-throughput microwave-based robotic platform (see Supplementary Note 2 for experimental setup).

We start our first set of experiments (first generation) which aims at giving the most diverse set of experimental synthesis conditions. We explored the following set of five variables:

1. Power of the microwave, by changing the power of the microwave we can influence the time it takes the reaction solution to reach the required temperature;
2. Solvent composition, our solution has a fixed composition: 80% water and 20% of organic solvent. The solvent composition was chosen to better solubilize all precursors (in particular porphyrin), which would help us achieve a high yield and crystallinity and minimize the amount of hazardous organic solvents used in the reactions. Preliminary results on the solvothermal synthesis of Al-PMOF showed more reproducible yields and good crystallinity with an 80% H_2_O:20% DMF solvent mixture (see Supplementary Note 3, Table S2 and Fig. S2). As solvents are deemed to be a critical factor in MOF synthesis as they can have a significant impact on the crystallization pathway and/or on the final product obtained^20,46^, we studied a total of five organic solvents with different boiling points (i.e., ethanol (EtOH), 1-propanol, dimethylformamide (DMF), dimethylacetamide (DMA) and dimethyl sulfoxide (DMSO)), which covered a wide range of temperatures from 75 to 190 °C. This provides an additional degree of flexibility and parameters to study in our work.
3. Reaction time, which is the total time our vial was in the microwave (including both: the time required to reach the temperature at which the reaction takes place (<1 min) and the reaction time itself);
4. Reaction temperature, the temperature at which the reaction is carried out;
5. Concentration of the reactants, the aluminum to porphyrin ratio was constant and set as in the hydrothermal synthesis^40^. Concentrations 1 and 2 possess the same amount of solvent but different amounts of precursors, while concentrations 2 and 3 possess the same amount of precursors but different volumes (see Supplementary Note 4 for experimental details). This systematic approach would allow us to assess the influence of both factors: concentration and pressure in the reaction vial.

The ranges of these variables were based on our experience with the solvothermal synthesis of Al-PMOF and are detailed in Table 1. In contrast to our previous work, where we treated solvents as a categorical variable described using a numeric array (so-called one-hot encoding in which the presence of a solvent is indicated with a 1 and absence with a 0), we describe solvents with their boiling points here. The boiling point is a critical factor when choosing solvents for a solvothermal synthesis^20^. Using a chemically motivated descriptor for solvents can help the machine learning model better interpolate between different solvent types, leading to better predictions and interpretations. Finally, for the new synthesis conditions suggested by the genetic algorithm, we choose the solvent with the closest boiling point.

**Table 1:** Experimental variables investigated in this study.

Variable	Range	Exploration weight
Power [W]	200–300	0.2
Temperature [°C]	175–200	0.7
Time [min]	20–60	0.7
Concentration [–]	1–3	0.8
Boiling point [°C]	80–190	1.0

The table shows the synthetic variables, ranges, and exploration weight factor, normalized to 1 for the most important variable (i.e., boiling point). The concentration was given discrete variables: 1, 2, and 3 corresponding to high, medium, and low concentration, respectively (see Supplementary Note 4 for experimental details).

### Design of the experimental protocols with the SyCoFinder

Based on the range of the variables given in Table 1, we used the SyCoFinder^39^ to generate a set of 25 most diverse experiments, which covers the space of experimental variables as widely as possible (see Supplementary Note 4, in particular, Table S4 for the synthesis conditions of samples from generation 1). To better initiate these experiments, we weighted the exploration of different synthesis variables with their importance quantified in our previous work using a machine learning model for the synthesis of HKUST-1^28^. Notably, in our previous work, we found that this chemical intuition (i.e., the importance of variables) is transferable to the synthesis of new materials. In addition, this chemical intuition matches our human chemical intuition based on our previous experiences with solvothermal synthesis. The weights for each variable are listed in Table 1. Hence, we assign the type of solvent (i.e., boiling point) as the most important variable. Reaction temperature, time, and concentration were thought to play a slightly less important role, and power the least important of all the variables studied (see the “Methods” section for the details of diverse set generation and the genetic algorithm).

The 25 reactions suggested by the SyCoFinder were then carried out utilizing the microwave and robotic platform (Fig. S1). After synthesis, each sample was collected individually by centrifugation, washed with the organic solvent used for the reaction itself, followed by acetone, and finally dried overnight in a ventilated oven at 60 °C. In some cases for which it seemed that some unreacted ligand was still present, DMF was also used. Working up the material with this type of solvent should help in the removal of unwanted products, in particular, the recrystallized porphyrin as it is more soluble. Moreover, the crystallinity of the materials should always be assessed via powder X-ray diffraction (PXRD) measurements to address the purity of the structure and avoid the presence of any additional phase.

### Crystalline structure and yield

**Fig. 2:**
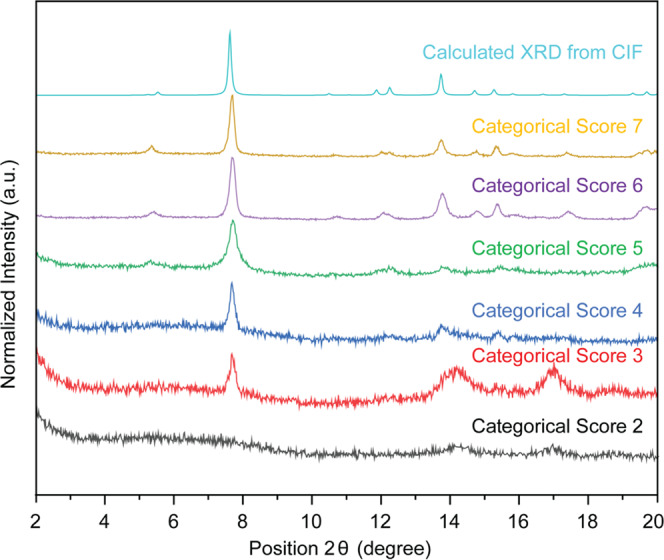
PXRD patterns of samples obtained in generation 1 for each categorical score ranging from 1 to 8. PXRD of the best and worst samples produced from the first generation of experiments, with the calculated XRD from the CIF of Al-PMOF. The categorical score was given on a scale from 1 (worst) to 10 (best) in terms of crystallinity.

The resulting reactions produce vastly different results; a number of experiments yielded little or no powder, and many experiments yielded amorphous products. The PXRD pattern was collected, showing very distinct crystallinity for the best and worst samples (Fig. 2). Seven reactions from the first generation yielded a PXRD pattern characteristic of Al-PMOF. The crystallinity was ranked on a scale of 1–10, where 1 was used for samples that did not yield a powder, 2–5 was for samples that were amorphous or had poor crystallinity, while higher numbers were given to powders that presented better crystallinity. Distinctions between 9 and 10 were made for those which presented additional peaks or fully matched the Al-PMOF predicted pattern without any additional phase or impurities, respectively.

Each PXRD pattern was analyzed individually and ranked qualitatively, depending on which pattern would match best the calculated XRD from the CIF of Al-PMOF. Evaluating the PXRDs by eye allows us to look at the spectrum as a whole rather than individual peaks and can give good insights into the crystallinity of the structure. Automatically ranking PXRD patterns using computer software could also be considered an objective measure that would allow for a more systematic analysis of the data obtained. However, it is highly complex to develop robust software which would take into account all experimental artifacts that could be misleading in some cases (e.g., amorphicity, unreacted ligand, etc.). Further work to assess the correspondence among PXRD patterns with a more accurate, systematic, and quantitative measure could be done. An example of how this could be implemented is thoroughly described in ref. ^47^.

**Fig. 3:**
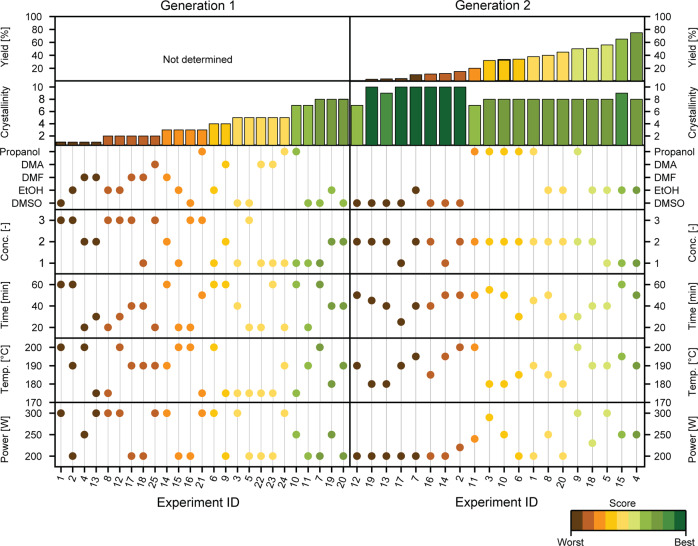
Parameters and results of optimization for each synthesis of generations 1 and 2. Each experimental variable studied (i.e., microwave power, reaction temperature, time, concentration, and solvents) selected for each Al-PMOF synthesis are depicted by circles, while the bar graphs illustrate the ranking of each reaction in terms of crystallinity and yield. The color code is given for the worst (brown) and best (dark green) samples. Generation 1 was ranked in terms of the crystallinity of each sample, while the success of generation 2 was determined by the yield as all samples proved to be highly crystalline. This proves the success of the GA in providing good crystallinity of all samples in just one generation. The PXRD patterns and N_2_ isotherms at 77 K can be visualized at https://www.cheminfo.org/flavor/zenodo/index.html?id=&id=7186602.

**Fig. 4:**
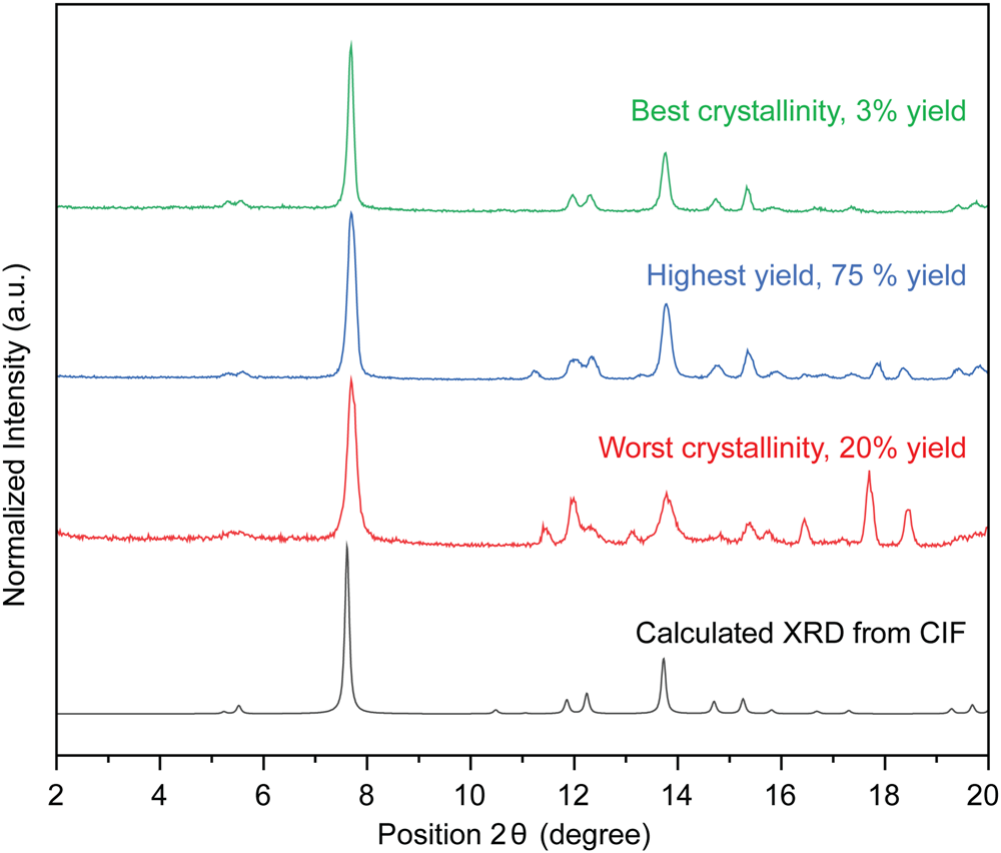
Best and worst PXRD patterns obtained in generation 2. PXRD of the best (highest crystallinity and yield) and worst crystallinity produced from the second generation of experiments, as well as the calculated XRD from the CIF of Al-PMOF.

The ranking from the first generation (Fig. 3) was used to further optimize the synthesis by generating the second generation of experiments with the genetic algorithm of SyCoFinder (Table S5). Again, after synthesis, the PXRD patterns were gathered and the experimental results were ranked. Interestingly, in this second generation, all of the material synthesized proved to be crystalline and matched the PXRD pattern of Al-PMOF (Fig. 4).

Our initial aim was to screen for both crystallinity and yield. As already after the first generation we obtained a near-perfect score on crystallinity, we could already rank our reaction conditions based on yield. An accurate calculation of the yield would require thermogravimetric analysis (TGA) and elemental analysis (EA). In this study, as a high-throughput approximation, we determined it by dividing the amount of powder obtained by the amount of porphyrin ligand used in the synthesis. Interestingly, a number of conditions gave excellent results, with a high yield and good crystallinity (Figs. 3 and 4).

For carbon capture applications it is important that the pore structure is the same as the one obtained via hydrothermal synthesis. As a high-throughput technique, we determined the surface area from a nitrogen (N_2_) isotherm at 77 K for the highest-ranked materials (samples 4 and 15 from generation 2, G2S4 and G2S15, respectively). From these isotherms (see Supplementary Note 5, Fig. S3), we obtained surface areas (1024 and 1226 m^2^ g^−1^, respectively) comparable to that previously reported with a hydrothermal synthesis (i.e., 1400 m^2^ g^−1^)^40^, which indicates it is likely that the robot synthesized material has retained the pore structure of the MOF, and so, it should be suitable for CO_2_ capture applications. This highlights the importance of the SyCoFinder in optimizing synthesis conditions. The methodology learns from failed and partially successful experiments and discards conditions that do not yield the desired product. A failed or successful synthesis is judged on criteria defined by the ultimate goal of the study: if one is seeking good crystallinity, high yield, or surface area (among other characteristics), a MOF that does not fulfill those requirements would be ranked worst and similar synthesis conditions would less likely be suggested in the following generations. This would lead us toward the completion of our goal of synthesizing a MOF with the characteristics of our interest. In our case, as the crystallinity and yield were sufficiently high and the surface area similar to what was previously obtained, there was no need for a third generation of experiments, as we would only expect minor improvements. Al-PMOF can thus be efficiently synthesized in a microwave by inserting AlCl_3_⋅6H_2_O (0.099 mmol, 24 mg) and TCPP (0.051 mmol, 40 mg) in a solution of H_2_O/EtOH (80%/20%) (2 mL). The vial is then sealed and inserted in the microwave for a 50 min reaction at 190 °C with 250 W of power.

### Reproducibility and large-scale MOF synthesis

The reproducibility of the highest-ranking synthesis condition (i.e., G2S4) was also tested with the robotic platform set up to run 16 reactions over a 24-h period. We synthesized five sets of 8 reactions each. After the syntheses, each set was combined into a single vial (i.e., batch) (see Supplementary Note 6 for experimental details). The powder was collected by centrifuge, and then washed with solvent and dried overnight. All PXRD patterns matched Al-PMOF (Fig. S4a), and the BET surface area and pore volume of the large sample determined from an N_2_ isotherm at 77 K were also comparable (i.e., 1264 and 0.628 cm^3^ g^−1^, respectively (Fig. S4b)). We then tested the microwave-synthesized material for CO_2_ capture and obtained an uptake of 3.5 mmol g^−1^ at 1 bar (Fig. S4c), which is very similar to the hydrothermally synthesized Al-PMOF (i.e., ca. 4 mmol g^−1^ at 1 bar). Continuously synthesizing using the platform this way can generate gram amounts of powder that can be used for further applications such as CO_2_ capture at a large scale.

For the large-scale MOF synthesis, since the reactions run sequentially one after the other, the first reaction mixtures are left at room temperature in the mother solution for very different times, whose longest could reach 16 h. It is therefore important to assess the stability of Al-PMOF in slightly acidic conditions for an extended amount of time as the hydrolyzed aluminum salt makes the solution slightly acidic. In its original publication^40^, it is demonstrated that this structure is stable under acidic solutions (i.e., pH = 5). Moreover, Oliver T. Wilcox, et al.^48^ have also reported Al-PMOF after loading it with different acids (hydrochloric acid (HCl) and formic acid) for 16 h, confirming the remarkable stability of the MOF under acidic conditions. We are therefore confident that leaving the MOF in the mother solution overnight would not have a large effect on its crystallinity and pore structure. For other MOFs, however, this may be a factor that should be considered. In the case of robotic synthesis, one possibility would be to automate a filtration and washing step of the sample after synthesis.

## Discussion

### Analysis of the experimental variables

**Fig. 5:**
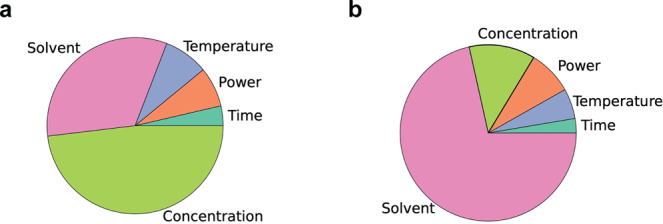
Pie charts showing the relative importance of each synthesis variable. **a** On crystallinity. **b** Yield. We use SHAP (SHapley Additive explanations) values to quantify the importance of variables. SHAP values quantify how each variable influences the outcome of the machine learning model using a game theoretic approach^56,57^.

In Fig. 3, we have summarized the results of this study and in Fig. 5 we show, through analysis of the failed and partially successful experiments, the relative importance of the experimental variables in obtaining (a) high crystallinity, and (b) high yield. From our analysis, we see that the changes in the concentration of reactants followed by changes in the solvent have the most impact on crystallinity. While for the yield, by far the most important criterion is the solvent type.

### Influence of the solvent

The standard hydrothermal procedure for the synthesis of this MOF shows that, although synthesized in pure water, a higher temperature (i.e., 180 °C) is required to dissolve the porphyrin and allow it to react with the aluminum precursor. Using a mixture of water and another organic solvent could help the porphyrin to dissolve, whilst retaining the high heat capacity of water which seems to be required to form the MOF. Solvothermal reactions with different H_2_O:DMF ratios (i.e., 20:80%, 50:50%, and 80:20%) were carried out (see Supplementary Note 3 for experimental details) and the optimal results were obtained with an 80:20% H_2_O:DMF ratio (Table S2 and Fig. S2). DMF is a common solvent for MOF synthesis^49^, due to its high dielectric constant and relatively high boiling point. It is interesting to look in some detail at the second generation of experiments that were proposed by SyCoFinder’s algorithm. In the first generation, DMF was included as an additional organic solvent, yet the second generation of reactions did not include any experiments with DMF. This is due to the fact that the crystallinity of samples with DMF is poor, and the other solvents yielded higher crystallinity. The analysis of the data shows that the solvent type, which we characterize by the boiling point, is one of the key variables that determine the crystallinity. The data also show that although the type of solvent is important, the quality of the crystals does not correlate with the boiling point. The yield may be better described by this factor: higher boiling point solvents (e.g., DMSO) show a much lower yield, while lower boiling point solvents (e.g., EtOH, 1-propanol) show a higher one (Fig. 3). This suggests that pressure favors the crystallization of the MOF. However, this may not be the only descriptor that correlates with the results. Future work on the influence of the solvent in the outcome of the reaction could be performed for a better understanding of the crystallization process of Al-PMOF. For a more detailed analysis, one would need to use multiple descriptors for the solvent (e.g., polarity, proticity, etc.) and run several experiments to accurately draw conclusive explanations.

### Influence of the concentration

The concentration of the precursors was also studied: Al-PMOF was obtained with the same metal-to-ligand ratio except for different amounts of solvent, which also leads to a change of the pressure inside the reaction vessel. As a control, concentrations 1 (high) and 2 (medium), possess the same volume but different amounts of metal and ligand, while concentration 3 (low), possesses the same amount of precursors as concentration 2 but a higher volume of solvent (Table S3). The analysis of the relative importance of experimental variables shows that concentration plays a major role in crystallinity. The lowest concentration (i.e., concentration 3, which also presents the largest amount of solvent, and thus the highest pressure) is not suggested in generation 2, as it leads to relatively poor crystallinity in generation 1. It seems that the combination of low concentrations and high pressures in the reaction vessel is not beneficial for the synthesis of Al-PMOF. On the other hand, if we compare concentrations 1 and 2, which possess the same volume, the highest concentration (i.e., concentration 1) tends to give better crystallinity overall, which may be positively correlated to the kinetics of the reaction^50^ (Fig. 3).

### Influence of other variables

The other variables studied (i.e., reaction time, temperature, and power of the microwave) were deemed to be less important for both analyses: crystallinity and yield of Al-PMOF synthesis (Fig. 5). These were adapted to our needs (i.e., low reaction time) and had been tuned according to our knowledge of the hydrothermal synthesis (i.e., reaction temperature), while the power was limited by our microwave reactor.

### Method applicability and translatability

MOF crystallization is a complex molecular process, and the synthesis recipes vary greatly from MOF to MOF. Therefore, it is important that our screening approach is versatile and easily adaptable to different synthesis optimization problems. The first step is to define the chemical space that we want to explore, which can be easily tuned according to our structure of interest, needs, and the variables that we want to optimize. Then, in contrast to the conventional “study one-factor-at-a-time”, here, the SyCoFinder generates the most diverse set of syntheses conditions, which interestingly leads chemists to reaction conditions that probably would have never explored otherwise. Then, GAs and ML iterate the data towards a successful synthesis which provides us with the best target that fulfills our requirements (e.g., good crystallinity, high yield, high surface area, etc.). This is simply a quantified “intuition” developed by the ML model, which is similar to the intuition developed by experienced chemists in the lab. The advantage here is that the software used in this study is open-access and available as a web application on the Materials Cloud^39^.

The translatability of the optimized microwave conditions into a conventional heating procedure was also investigated. Al-PMOF was therefore solvothermally synthesized with the conditions that yielded the best-ranked material of generation 2 (i.e., G2S4, see Supplementary Note 7 for experimental details). The yields obtained ranged from 50% to 60%. Similar to the microwave synthesis, also here the yield was calculated by dividing the amount of MOF powder obtained by the amount of TCPP ligand used in the synthesis. The PXRD pattern (Fig. S5) confirms the crystallinity of the structure as it fully matches the calculated XRD from the CIF. The successful synthesis of Al-PMOF, along with the relatively high yield obtained, confirms the adaptability of the microwave-optimized parameters to a different type of MOF synthesis equipment. This demonstrates the success of SyCoFinder in optimizing synthetic conditions and the applicability of the method to different materials and equipment.

Similarly, the microwave synthesis of Al-PMOF in pure water was also tested. The conditions used correspond to the best one obtained from generation 2 (i.e., G2S4). However, instead of an H_2_O:EtOH solvent mixture, pure water was used (see Supplementary Note 8 for experimental details). The PXRD was crystalline and matched the calculated XRD from the CIF of Al-PMOF (Fig. S6). However, the reaction yield was low and out of the three reactions performed, it only reached a maximum of 13% yield. These results suggest that the use of a co-solvent strongly helps in the synthesis of Al-PMOF with high yields.

## Conclusions

In summary, we have developed an alternative Al-PMOF synthesis method, using a microwave reactor with comparable crystallinity, surface area, and CO_2_ uptake to the traditional hydrothermal synthesis of Al-PMOF, but with a higher yield and a much shorter reaction time.

The other interesting part of this work is the methodology that we used to find the optimal synthesis conditions: an experimental design that learns from failed and partially successful experiments. Although we used a robot in this work, the total number of experiments that were used to find these conditions, only two generations and a total of 45 reactions, illustrate that the underlying methodology does not require very large data sets to be of practical use.

We hope that our results encourage authors to publish their failed and partially successful experiments. The fact that we only publish the successful recipes creates a bias in the literature, that makes predictions of the reaction conditions using machine learning more difficult^51^. Of course, in our case, as we are using a robot, publishing the failed and partially successful conditions in addition to the successful recipe does not create an additional burden. Jablonka et al.^51^ outline some ideas on how the burden of reporting all experimental results can be minimized.

## Methods

### Synthesis Condition Finder (SyCoFinder)

The synthesis condition-finding procedure is adapted from our previous work^28^. In this procedure, we initiate our experiments with a generation of the most diverse set of experiments identified using the farthest point sampling (i.e., MaxMin diversity). In this approach, to come up with *N* trials, we first add a trial chosen randomly. Then, for the other *N*−1 trials, we iteratively add the most dissimilar synthesis conditions to the set of previously selected trials, where we maximize the minimum distance to the currently selected trials. Here, the dissimilarity metric is the euclidean distance between two synthesis conditions weighted with an exploration factor that is listed in Table 1. Synthesis variables with higher weight are explored more.

After this first generation, we use a genetic algorithm (GA), which is a global optimization algorithm, to explore the synthesis conditions space that we identified in Table 1. The GA uses genetic operations, including selection, crossover, and mutation, to generate new offspring from the previous generations. In this approach, two trials from the previous generation (parents) are selected, and their synthesis variables (genes) are combined using a crossover operation to generate a new synthesis trial (offspring). To include a chance to explore beyond the previous generations, some of the genes can mutate. The ratio between crossover and mutation balances the exploration vs. exploitation for the optimization. The synthesis trials with higher scores have a higher chance of being selected to transfer their genes to the next generation. As we use a ranking-based selection algorithm, the score function can be easily adapted to any target, e.g., crystallinity, yield, etc. The details of the genetic algorithm, including the crossover and mutation and the diverse set computations, are reported in our previous work^28^.

### Chemical synthesis

Detailed synthesis conditions for each Al-PMOF reaction performed in this study can be found in the Supplementary Information (see Supplementary Note 4, Tables S4 and S5). The syntheses were carried out in a microwave synthesis reactor (Biotage, Uppsala, Sweden) which is connected to a high-throughput robotic platform (Chemspeed technologies, Füllinsdorf, Basel, Switzerland) (Fig. S1). The microwave is completely automated and executed with the Chemspeed autosuite software. All chemicals were purchased from commercial sources and used without further purification.

### Characterization

Powder X-ray diffraction (PXRD) patterns of all samples were collected on a Bruker D8 Advance diffractometer at ambient temperature using monochromated Cu K*α* radiation (*λ* = 1.5418 Å), with a 2*θ* step of 0.02° with different 2*θ* ranges. Simulated PXRD patterns were generated from the corresponding crystal structures using Mercury 3.0.

The N_2_ adsorption isotherm measurements were performed at 77 K using a BELSORP Mini (BEL Japan, Inc.). Prior to measurements, samples were activated at 180 °C for 12 h under dynamic vacuum. The N_2_ adsorption isotherm in the *p*/*p*_0_ range 0.06–0.25 was fitted to the Brunauer–Emmett–Teller (BET) equation to estimate the surface area of the samples.

The CO_2_ isotherm at 298 K was collected by a gravimetric method using an IGA system (Intelligent Gravimetric Analyser, Hiden Isochema Ltd., Warrington, UK).

## Supplementary information

Smit_PR File

Supplementary Information

## Data Availability

All data generated during this study are included in this article and respective Supplementary Information. The characterization data (including powder X-ray diffraction (PXRD) patterns, N_2_ and CO_2_ isotherms at 77 and 298 K, respectively) are saved in the electronic lab notebook (ELN)^51–53^. The spectra are usually stored in JCAMP-DX format and the sample information with metadata in JavaScript Object Notation (JSON). The characterization data that supports the findings of this study are available on Zenodo (10.5281/zenodo.7186602)^54^ and can be visualized through the following view developed with the visualizer library: https://www.cheminfo.org/flavor/zenodo/index.html?id=&id=7186602^55^.

## Code availability

The genetic algorithm and diverse set sampling codes are available via https://www.materialscloud.org/work/tools/sycofinder.
