# Supplementary material for: Using genetic algorithms to systematically improve the synthesis conditions of Al-PMOF
Source: Commun Chem. 2022 Dec 10;5:170. doi: 10.1038/s42004-022-00785-2 (PMC9814730; doi:10.1038/s42004-022-00785-2)
Supplement: Supplementary file 2 — Supplementary Information [file 42004_2022_785_MOESM2_ESM.pdf]

Supporting Information: Using genetic algorithms to systematically improve the synthesis conditions of Al-PMOF

Nency P. Domingues,[1] Seyed Mohamad Moosavi,[1,2] Leopold Talirz,[1,3] Kevin Maik Jablonka,[1] Christopher P. Ireland,[1] Fatmah Mish Ebrahim,[1,4] and Berend Smit*,[1]

1*Laboratory of Molecular Simulation (LSMO), Institut des Sciences et Ingénierie Chimiques, École Polytechnique Fédérale de Lausanne (EPFL), Sion, Valais, Switzerland.*

2*Department of Mathematics and Computer Science, Freie Universität Berlin, Berlin, Germany.*

3*Theory and Simulation of Materials (THEOS), School of Engineering (STI), École Polytechnique Fédérale de Lausanne (EPFL), Lausanne, Vaud, Switzerland.*

4*Cavendish Laboratory, School of Physical Sciences, University of Cambridge, Cambridge, United Kingdom.*

E-mail: berend.smit@epfl.ch

Contents

Supplementary Note 1: Hydrothermal Reactions - Variable Yield **S-3**

Supplementary Note 2: Robotic Platform **S-4**

Supplementary Note 3: Solvothermal Reactions - Optimal H_2O:DMF Ratio **S-5**

Supplementary Note 4: SyCoFinder: Microwave Syntheses **S-7**

Supplementary Note 5: N_2 Isotherms of the Highest Ranked Materials **S-11**

Supplementary Note 6: Reproducibility and Large-Scale MOF Synthesis **S-12**

Supplementary Note 7: Method Versatility - Solvothermal Synthesis **S-14**

Supplementary Note 8: Method Versatility - Microwave Synthesis in Pure H_2O **S-16**

References **S-17**

Supplementary Note 1: Hydrothermal Reactions - Variable Yield

The hydrothermal synthesis of Al-PMOF[S1] gives variable yield. Table S1 summarizes the yield of four different Al-PMOF syntheses under hydrothermal conditions.

Table S1: Hydrothermal reaction syntheses presenting variable yield ranging from 38 and 88% . The yield was estimated by dividing the amount of MOF powder obtained by the amount of porphyrin ligand used in the synthesis.

Hydrothermal Reaction	Temperature [°C]	Time [h]	Yield [%]
**1**	180	16	65
**2**			88
**3**			80
**4**			38

The reactions were performed as follows: a mixture of $AlCl_3 \cdot 6\,H_2O$ (0.124 mmol, 30 mg) and the free-base meso-tetra(4-carboxyphenyl)porphine (H_2TCPP) (0.063 mmol, 50 mg) was suspended in 5 mL of deionized water in a 23 mL Teflon-lined autoclave. The mixture was sonicated for 10 minutes at room temperature and then heated up to 180 °C for 16 hours. The heating and cooling rates are 2 °C/min and 1.5 °C/min, respectively. The product was washed 3 times with DMF and once with acetone.

Supplementary Note 2: Robotic Platform

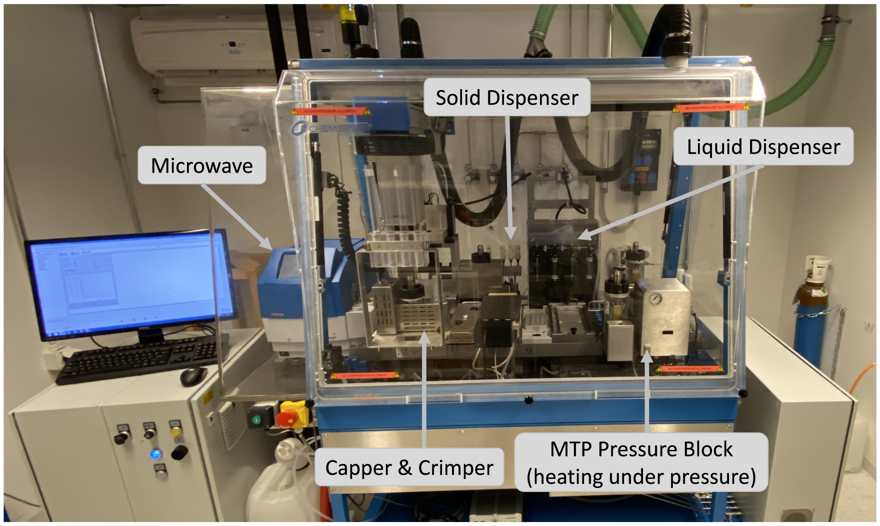

Figure S1: Robotic platform used for the synthesis of Al-PMOF.

Supplementary Note 3: Solvothermal Reactions - Optimal H_2O:DMF Ratio

The hydrothermal synthesis of Al-PMOF[S1] gives variable yield. Different ratios of water and DMF were studied to assess the influence of the solvent ratio on the crystallinity of the MOF as well as the yield of the reaction. The ratios investigated are shown in Table S2.

Table S2: Solvothermal reaction syntheses presenting variable H_2O:DMF ratios with the resulting yields. The yield was estimated by dividing the amount of MOF powder obtained by the amount of porphyrin ligand used in the synthesis.

**Solvothermal Reaction**	**Temperature [°C]**	**Time [h]**	**H_2O:DMF [%]**	**Yield [%]**
**1**			20:80	0
**2**	180	16	50:50	49
**3**			80:20	74

The reactions were performed as follows: a mixture of $AlCl_3 \cdot 6\,H_2O$ (0.124 mmol, 30 mg) and H_2TCPP (0.063 mmol, 50 mg) was suspended in different ratios of H_2O and DMF in a 23mL Teflon-lined autoclave (Table S2). The mixture was sonicated for 10 minutes at room temperature and then heated up to 180 °C for 16 hours. The heating and cooling rates are 2 °C/min and 1.5 °C/min, respectively. The product was washed 3 times with DMF and once with acetone. The PXRD patterns were measured for each of the samples. The solvothermal reaction 1 (H_2O:DMF (20:80%)) did not yield any powder while reactions 2 and 3 resulted in the characteristic Al-PMOF PXRD pattern (Figure S2).

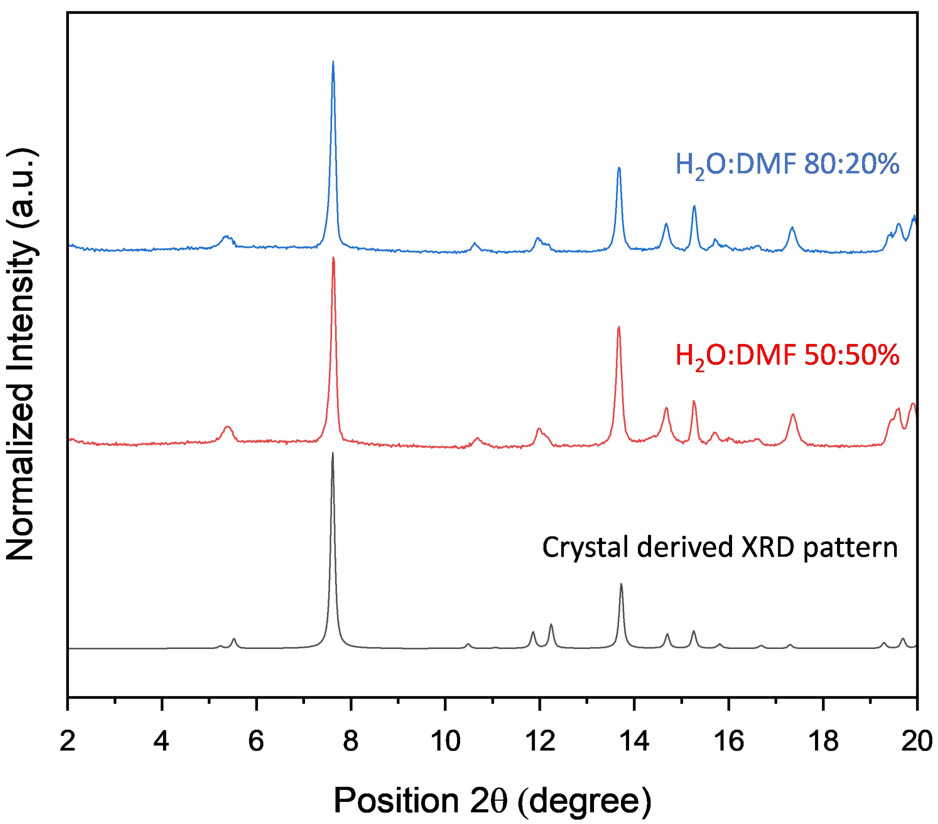

Figure S2: PXRD patterns of the solvothermal reactions 2 and 3 with H_2O:DMF ratios of 50:50% and 80:20%, respectively, including the calculated XRD from the CIF of Al-PMOF. Reaction 1, with a H_2O:DMF ratio of 20:80% did not yield any powder.

Considering the relatively high yield and good crystallinity of the samples with 50:50% and 80:20% H_2O:DMF ratio, the latter was chosen for the optimization of the synthesis in the microwave as it contained a larger amount of water.

Supplementary Note 4: SyCoFinder: Microwave Syntheses

Two generations of experiments were designed by the SyCoFinder. Each reaction condition is given in detail in Tables S4 and S5 for generation 1 and 2, respectively. The power of the microwave [W], reaction time [minutes] and temperature [°C], concentration and type of organic solvent used in the synthesis (80% H_2O - 20% organic solvent) are given in each table.

Three discrete variables are used to describe the concentrations studied: 1, 2 and 3, corresponding to high, medium and low concentration. The total amounts of precursors (i.e., metal ($AlCl_3 \cdot 6\,H_2O$) and H_2TCPP and total volume of solvent (consisting of 80% H_2O and 20% of organic solvent) are shown in Table S3. The metal to ligand ratio was kept constant while the volume of solvent was changed from 2 to 4 mL. The synthesis procedure is the following: a mixture of $AlCl_3 \cdot 6\,H_2O$ and H_2TCPP is inserted in the respective H_2O: organic solvent mixture. It is sonicated for 10 minutes at room temperature and taken to the robot tray for the reaction. Each reaction condition performed in this study is detailed in Tables S4 and S5, , for generations 1 and 2, respectively. The amount of precursors used can be found in Table S3).

Table S3: Respective amounts of precursors used for the synthesis of Al-PMOF.

**Concentration**	V_{TOT} [mL]	TCPP	$AlCl_3 \cdot 6H_2O$
**1**	2	0.051 mmol (40 mg)	0.099 mmol (24 mg)
**2**	2	0.025 mmol (20 mg)	0.050 mmol (12 mg)
**3**	4	0.025 mmol (20 mg)	0.050 mmol (12 mg)

Generation 1 (Table S4) explores the most diverse set of experimental conditions for the synthesis of Al-PMOF. For this generation, a categorical score is given to each sample according to its crystallinity. 1 is attributed to conditions which did not yield a powder, 2-5 is for samples that were amorphous or had poor crystallinity, while higher numbers are given to powders which present better crystallinity.

In generation 2 (Table S5), all samples proved to be crystalline, so the yield is also

investigated (determined by weighing the powder obtained divided by the amount of porphyrin ligand used in the synthesis). The categorical score is also attributed depending on the crystallinity of the samples.

Table S4: Microwave reaction conditions for samples of generation 1. The main goal of this first generation is to synthesize Al-PMOF in the microwave. The categorical score is given based on the crystallinity of each sample and ranged from 1 (worst crystallinity/no powder obtained) to 10 (best crystallinity). The PXRD patterns can be visualized through `https://www.cheminfo.org/flavor/zenodo/index.html?id=&id=7186602`.

Sample	Power [W]	Temperature [°C]	Time [min]	Concentration [-]	Solvent	Categorical Score
**G1S1**	300	200	60	3	DMSO	1
**G1S2**	200	190	60	3	EtOH	1
**G1S3**	300	175	40	1	DMSO	5
**G1S4**	250	200	20	2	DMF	1
**G1S5**	200	175	20	3	DMSO	5
**G1S6**	300	200	60	1	EtOH	4
**G1S7**	200	200	60	1	DMSO	8
**G1S8**	300	175	20	3	EtOH	2
**G1S9**	200	175	60	2	DMA	4
**G1S10**	250	175	60	1	Propanol	7
**G1S11**	200	190	20	1	DMSO	7
**G1S12**	300	200	30	3	EtOH	2
**G1S13**	300	175	30	2	DMF	1
**G1S14**	300	190	60	2	DMF	3
**G1S15**	200	200	20	1	EtOH	3
**G1S16**	200	200	20	3	DMSO	3
**G1S17**	200	190	40	3	DMF	2
**G1S18**	200	190	40	1	DMF	2
**G1S19**	250	180	40	2	EtOH	8
**G1S20**	200	190	40	2	DMSO	8
**G1S21**	300	175	50	3	Propanol	3
**G1S22**	200	175	20	1	DMA	5
**G1S23**	200	175	60	1	DMA	5
**G1S24**	300	190	20	1	Propanol	5
**G1S25**	300	190	20	3	DMA	2

Table S5: Microwave reaction conditions for samples of generation 2. Considering that Al-PMOF was obtained with good crystallinity for all samples of this generation, the yield was also estimated by dividing the amount of MOF powder obtained by the amount of porphyrin ligand used in the synthesis. The categorical score was given based on the crystallinity of each sample and ranged from 1 (worst crystallinity/no powder obtained) to 10 (best crystallinity). The PXRD patterns and N_2 isotherms at 77 K can be visualized through `https://www.cheminfo.org/flavor/zenodo/index.html?id=&id=7186602`.

Sample	Power [W]	Temperature [°C]	Time [min]	Concentration [-]	Solvent	Categorical Score	Yield [%]
**G2S1**	200	190	45	2	Propanol	8	38
**G2S2**	220	200	50	2	DMSO	10	15
**G2S3**	290	180	55	2	Propanol	8	32
**G2S4**	250	190	50	1	EtOH	8	75
**G2S5**	300	190	40	1	EtOH	8	56
**G2S6**	200	185	30	2	Propanol	7	34
**G2S7**	200	195	40	2	EtOH	10	10
**G2S8**	250	185	50	2	EtOH	8	40
**G2S9**	300	200	30	2	Propanol	8	50
**G2S10**	250	180	50	2	Propanol	8	33
**G2S11**	240	200	50	2	Propanol	7	20
**G2S12**	200	190	50	2	DMSO	7	1.0
**G2S13**	200	180	40	2	DMSO	9	3.5
**G2S14**	200	195	50	1	DMSO	10	12
**G2S15**	250	195	60	1	EtOH	9	65
**G2S16**	200	185	40	2	DMSO	10	11
**G2S17**	200	190	25	1	DMSO	10	4.0
**G2S18**	230	190	40	2	EtOH	8	51
**G2S19**	200	180	45	2	DMSO	10	3.0
**G2S20**	200	180	30	2	EtOH	8	45

Supplementary Note 5: N_2 Isotherms of the Highest Ranked Materials

The N_2 isotherms at 77 K for the most crystalline materials from generation 2: samples 4 and 15 (i.e., G2S4 and G2S15, respectively), are shown in Figure S3. The BET surface area correspond to 1024 and 1226 m^2g^{-1}, while the pore volumes are 0.687 and 0.736 cm^3g^{-1}, respectively.

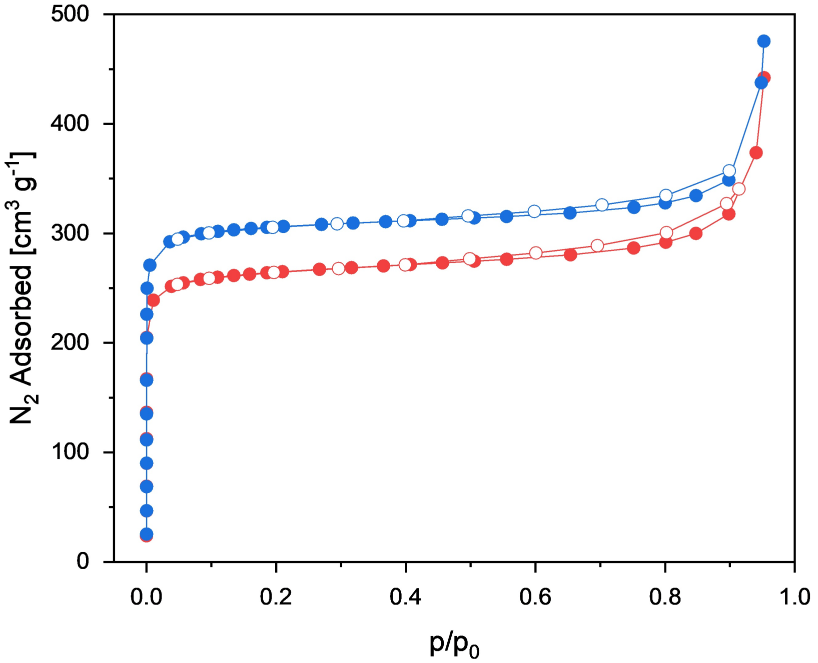

Figure S3: Single-component adsorption (filled) and desorption (open) isotherms for N_2 collected on activated Al-PMOF at 77 K. The red and blue curves correspond to samples 4 and 15 of generation 2, respectively.

Supplementary Note 6: Reproducibility and Large-Scale MOF Synthesis

For the large-scale Al-PMOF synthesis, the conditions that yielded the best results in generation 2 were used (i.e., G2S4). The procedure was as follows: a mixture of $AlCl_3 \cdot 6H_2O$ (0.099 mmol, 24 mg) and TCPP (0.051 mmol, 40 mg) was inserted in a solution of H_2O/EtOH (80%/20%) (2 mL). The vial was then sealed and inserted in the microwave for a 50 minutes reaction at 190 °C with 250 W of power.) We synthesized five sets of 8 reactions each. After the syntheses, each set was combined into a single vial and the PXRD patterns were measured as shown in Figure S4 a). Once the crystallinity of all 5 sets had been assessed, these were further combined into a larger batch whose yield was approximately 68%. The N_2 isotherm at 77 K of the combined batch was measured providing a BET surface area of 1264 $cm^3\ g^{-1}$ and pore volume of 0.628 $cm^3\ g^{-1}$ (Figure S4 b)). The CO_2 uptake was then measured after solvent exchange with acetone for 24 hours and activating the structure at 180 ° C under dynamic vacuum (Figure S4 c)). The uptake of 3.5 mmol/g at 1 bar is very close to the one reported in[S2] (i.e., 4 mmol/g), which had been synthesized hydrothermally. This confirms that the synthesis of Al-PMOF in a microwave as we describe in this study provides similar pore structure as conventional heating and gives similar N_2 and CO_2 adsorption results.

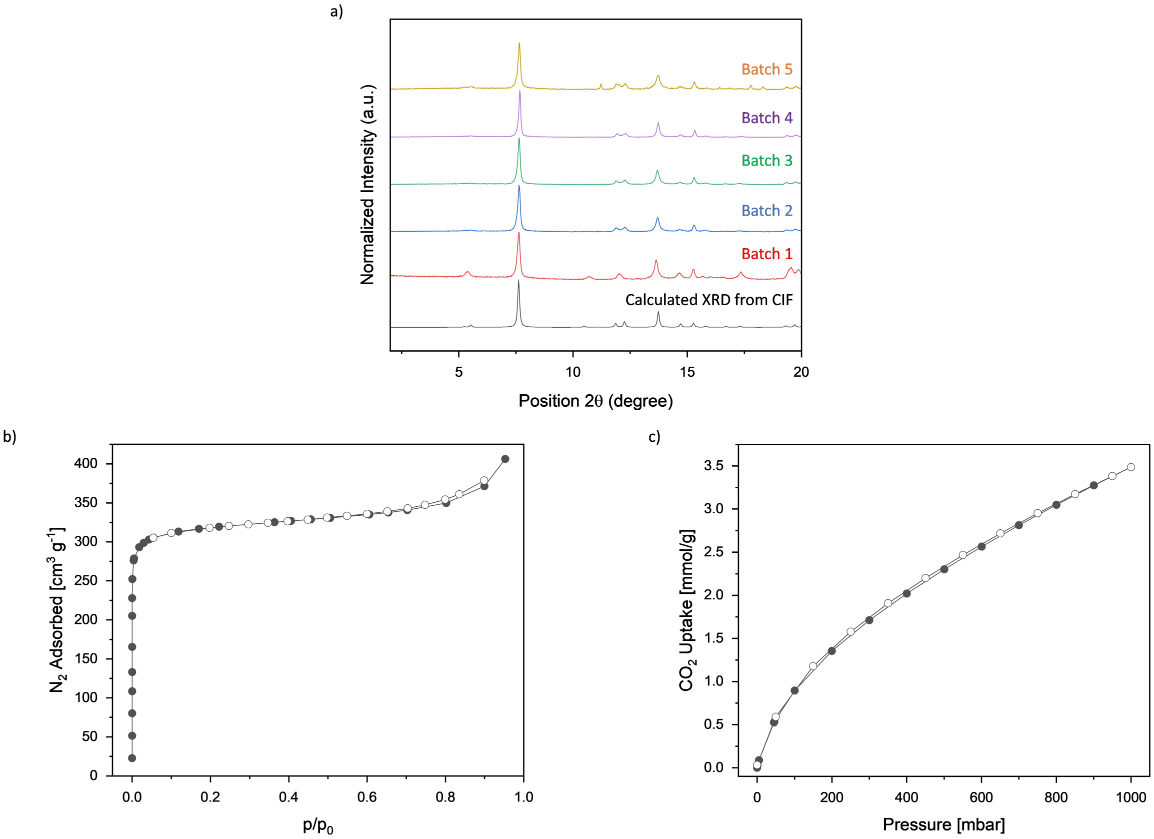

Figure S4: a) PXRD of all five batches of Al-PMOF synthesized in the robot; b) N_2 and c) CO_2 isotherms of the combined batches at 77 K and 298 K, respectively. Adsorption (filled) and desorption (empty) circles.

Supplementary Note 7: Method Versatility - Solvothermal Synthesis

The translatability of the optimized microwave conditions into a conventional heating procedure was also investigated. Figure S5 presents the PXRD pattern obtained. The time parameter was adapted and set the same as in the original hydrothermal synthesis (i.e., 16 hours). The amount of precursors was scaled-up to the 23 mL Teflon-lined bomb used: $AlCl_3 \cdot 6H_2O$ (0.23 mmol, 55.2 mg) and TCPP (0.12 mmol, 92 mg) were mixed in 4.6 mL of a solvent mixture (80% H_2O:20% EtOH) and sealed in a 23 mL Teflon-lined bomb. The reaction was performed at 190 ° C for 16 hours with a heating and cooling rates of 2 and 0.2 °C/min, respectively. The PXRD pattern (Figure S5) confirms the crystallinity of the structure as it fully matches the calculated XRD from the CIF.

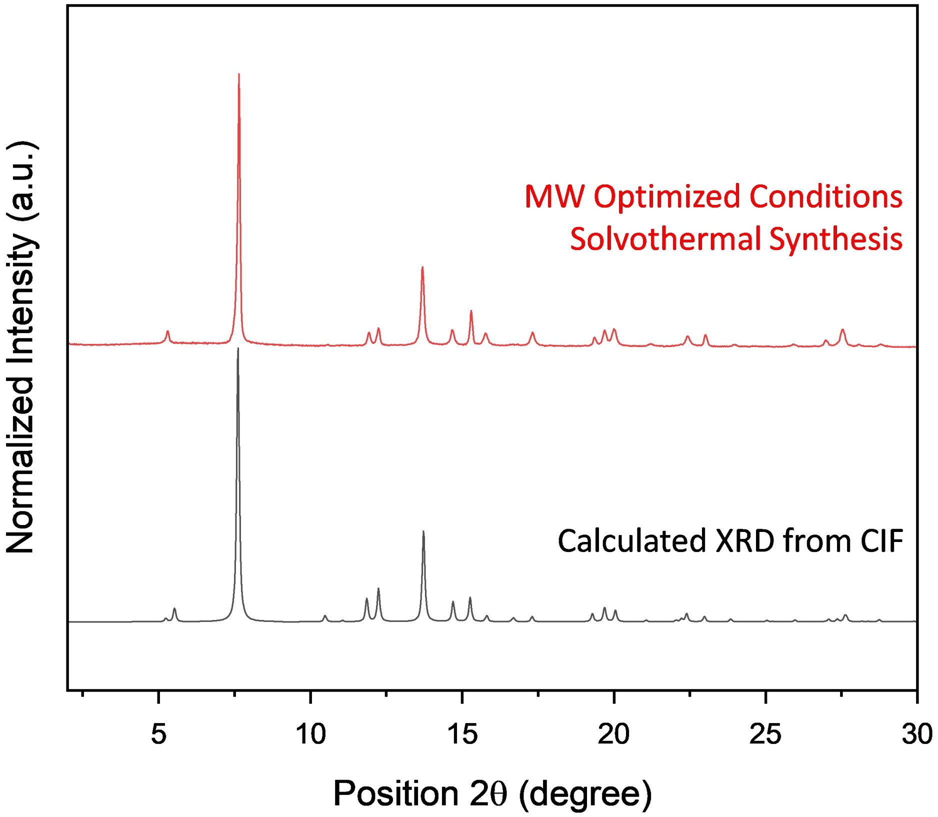

Figure S5: PXRD patterns obtained for the best performing microwave conditions performed in a solvothermal reaction as well as the calculated XRD from the CIF of Al-PMOF.

Supplementary Note 8: Method Versatility - Microwave Synthesis in Pure H_2O

The pure water microwave synthesis was performed as follows: a mixture of $AlCl_3 \cdot 6H_2O$ (0.099 mmol, 24 mg) and TCPP (0.051 mmol, 40 mg) was inserted in ultra-pure H_2O (2 mL). The vial was then sealed and inserted in the microwave for a 50 minutes reaction at 190 °C with 250 W of power.

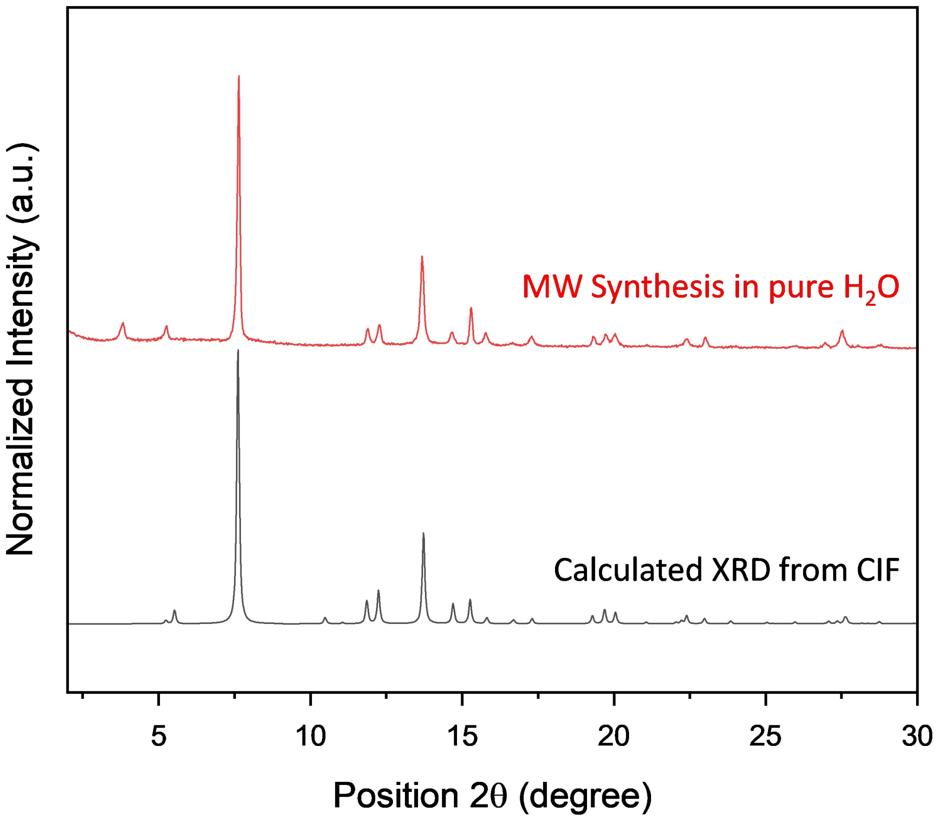

Figure S6: PXRD for the microwave synthesis in pure water and the calculated XRD from the CIF of Al-PMOF.

References

(S1) Fateeva, A.; Chater, P. A.; Ireland, C. P.; Tahir, A. A.; Khimyak, Y. Z.; Wiper, P. V.; Darwent, J. R.; Rosseinsky, M. J. A water-stable porphyrin-based metal-organic framework active for visible-light photocatalysis. *Angewandte Chemie International Edition* **2012**, *51*, 7440–7444.

(S2) Boyd, P. G.; Chidambaram, A.; García-Díez, E.; Ireland, C. P.; Daff, T. D.; Bounds, R.; Gładysiak, A.; Schouwink, P.; Moosavi, S. M.; Maroto-Valer, M. M., et al. Data-driven design of metal–organic frameworks for wet flue gas CO2 capture. *Nature* **2019**, *576*, 253–256.
