# Supplementary material for: Using genetic algorithms to systematically improve the synthesis conditions of Al-PMOF
Source: Commun Chem. 2022 Dec 10;5:170. doi: 10.1038/s42004-022-00785-2 (PMC9814730; doi:10.1038/s42004-022-00785-2)
Supplement: Supplementary file 1 — Smit_PR File [file 42004_2022_785_MOESM1_ESM.pdf]

nature portfolio

Peer Review File

Using genetic algorithms to systematically improve the synthesis conditions of Al-PMOF

Open Access This file is licensed under a Creative Commons Attribution 4.0 International License, which permits use, sharing, adaptation, distribution and reproduction in any medium or format, as long as you give appropriate credit to the original author(s) and the source, provide a link to the Creative Commons license, and indicate if changes were made. In the cases where the authors are anonymous, such as is the case for the reports of anonymous peer reviewers, author attribution should be to 'Anonymous Referee' followed by a clear attribution to the source work. The images or other third party material in this file are included in the article's Creative Commons license, unless indicated otherwise in a credit line to the material. If material is not included in the article's Creative Commons license and your intended use is not permitted by statutory regulation or exceeds the permitted use, you will need to obtain permission directly from the copyright holder. To view a copy of this license, visit http://creativecommons.org/licenses/by/4.0/.

Reviewers' comments:

Reviewer #1 (Remarks to the Author):

The review is provided in tha attachement file below.

Reviewer #2 (Remarks to the Author):

In their work, Domingues and coworkers report the use of a genetic algorithm to find better synthetic conditions for a promising MOF for CO2 capture, Al-PMOF. Various reaction conditions are varied through the genetic algorithm including temperature, reaction time, concentration, and solvent boiling point. It's a nice example and extension of the previous HKUST-1 work but applied to a different MOF with less synthetic data available. The insights provided on the effects from the different synthetic variables on the figures of merit of yield and crystallinity are interesting. It showcases the GA approach as a viable methodology to study synthesis conditions for MOFs on a larger scale. Overall, the manuscript contains enough details except for how solvent is treated in the GA. The authors mention that one-hot encoding was used previously but now they describe the solvents with a continuous variable. It is unclear how that was done. They could have meant that it was strictly the boiling point of the solvent, but that also seems strange since they only include five different solvents each with its own boiling point, so it's not really continuous. This point needs to be made clearer. Once that is done, I think the manuscript is ready for publication.

Reviewer #3 (Remarks to the Author):

See separate file

Review

Using genetic algorithms to systematically improve the synthesis conditions of Al-PMOF

The authors present a joint machine learning and experimental approach to optimize the synthesis conditions of Al-PMOF. This work is pretty original for the field of synthetic chemistry, as well as the use of algorithms towards synthetic chemists, the encouragement to communicate upon the failed experiments to improve knowledge and processes is also a relevant point for the community.

The idea and work developed in the manuscript are original and of true interest for the community of MOF chemists. Still, the work is sometimes explained too rapidly and some important data are not presented. Therefore, I encourage the authors to improve their manuscript prior to publication in Communications Chemistry.

Regarding the main manuscript, the following points are to be corrected/addressed in the text:

-In the introduction, the discussion about the binding strength is confusing, as the concepts of kinetics and thermodynamics are mixed. This should be better explained.

-The authors mention only one synthesis condition reported to date (hydrothermal, high temperature). This should be balanced as there have been several reports where Al-PMOF could be obtained from alumina precursors in DMF/water mixtures at lower temperatures and in very comparable solvents ratios presented in this manuscript. See the publications of G. Parsons on this matter (ex: https://doi.org/10.1016/j.matt.2019.11.005). I think it is worth mentioning these examples.

-When discussing the low yield, the working up difficulties should be mentioned, especially to get rid of unreacted species in MOF types materials.

Results:

-An important concern to me: from the manuscript and the SI it remains not clear to me why pure water was not included as a possible solvent for microwave tests? In fact, the authors test it with conventional heating and report reproducibility issues. They then test H2O/DMF with conventional heating and report and optimized 80/20 ratio. It is very confusing why neither pure water nor H2O/DMF mixture was tested in microwave sets of experiments? The paragraph 2. At page 4 does not provide any evidence on why these solvents are not tested. Please include these in the microwave test reactions and/or properly discuss why not.

-Yields such a 90% in pure water seem very doubtful to me given the low solubility of the ligand in water and more importantly the fact that the reactions are performed in excess of porphyrin (therefore the max possible yield is below 100%).

-page 5 “one-hot encoding for solvent type” what does this mean?

-Regarding the solvents, why only the boiling point is considered.? I think that much deeper discussion/work on solvent influence could be done. I guess the protic/aprotic type as well as the polarity are not less important matters.

-The concept of “our chemical intuition” is not very clear. How would the intuition form one chemist to another impact on the whole study?

-In Figure 1, “crystal derived XRD pattern” and “predicted” are not the right formulations and could induce in error thinking that the Al-PMOF structure was solved from single crystal diffraction when it was solved from HR-PXRD data. The pattern is calculated from the cif file and not predicted.

This figure should be changed to be much more informative. Please make a figure with much more patterns (at least one pattern per score from 1 to 8 for generation 1), showing the scores of different experiments so the crystallinity evolution along the scores become clearly visible. This way the phase purity/impurity will also be visible.

-Please state clearly the best conditions obtained after the 2 generations run.

-Page 7: the authors say there was no need for a 3rd generation. I would still want them to discuss what would be the possibilities of a 3rd run? Would it be judicious/possible to introduce a new parameter from one generation to another based on the best performing conditions rather to optimize the same parameters?

-Figure 2: should be more informative; provide the corresponding exact yield for each pattern.

-Page 8: hydrothermal instead of solvothermal

The experimental data for the N2 sorption isotherms should be provided.

-Reproducibility and large MOF synthesis: the word “scale” is missing (the MOF isn’t large).

On the matter of large-scale synthesis experimental data are lacking. Please provide quantitative data and results in terms of yield obtained for large scale, exact mas of material, the N2 and CO2 sorption experiments should be provided (in the SI).

Rather then multiplying the reactions, is it possible to perform the same reaction with the same yield and crystallinity in a larger reactor?

The study presents very nice results stating that the synthesis could be performed in a less toxic solvent in a higher yield. For the sake of synthesis speed the authors switched from hydrothermal to microwave synthesis. It would be of interest to check whether the microwave optimized conditions could be transposed to conventional heating procedure. The time parameter will certainly be increased but it is an important matter to discuss in terms of the adaptability of the optimized parameters to different kinds of synthesis equipment.

-Discussion:

In the first synthesis describe the figures in their order or change the order of figures.

-While performing microwave reactions with the assistance of robotic set up is very handy, I have a concern about the order of the reactions being tested. Indeed, 25 reactions are performed in the first run. This means that between the 1st and the last reactions the mixtures are left at room temperature for very different times (over 10 hours). This could have an impact on the reaction outcome. Please consider this parameter and comment whether this is a variable that impacts the synthesis. For how long can the mixture be left standing without noticeable impact?

Supporting Information

Page S3: “was dissolved” is not appropriate, a suspension would for given the low solubility.

Please indicate the moles for each reactant rather than just the mass.

Given the low scale of the reactions run, is it really possible to deduce the yield with a precision below 1% (for ex 3.5 % in table S5).

Referee report on<br><br>Using Genetic algorithms to systematically improve the synthesis conditions of Al-PMOF<br><br>By Smit et al.

The work of Smit et al. is a very interesting and original contribution, showing a methodology to systemically find the synthesis conditions for Metal Organic Frameworks that yield materials with high crystallinity and yield. The work builds further on their earlier contributions in the field. The methodology aims to systematically perform synthesis of materials in a directed way, in contrary to the trial and error approach that is commonly used. The work is an interesting contribution to the MOF field and might stimulate researchers to also publish their failed experiments. The work deserves to be published after some clarifications have been made as detailed below.

General comment : To improve the impact of the work for the broader scientific community, it is advised that the authors comment on the general applicability of the method, in how far it is usable for other researchers in the field. Furthermore, it is suggested that the authors motivate better why they used the particularly chosen MOF and elaborate on the general applicability to a broader set of MOFs and other synthesis protocols.

More detailed comments :

It is advised to introduce the material under investigation “Al-PMOF” better, show the chemical structure of the building blocks and the structure of the finally obtained material. It is suggested to insert this clarification in the introduction, when the material is first mentioned.

The authors used a high-throughput microwave-based robotic platform to test various parameters of the synthesis conditions. On page 5, it is mentioned that the SyCoFinder was used to generate the 25 most diverse experiments, however this statement is not fully clear based on the information given in the paper. The SyCoFinder methodology should be better introduced. It is advised to refer better to the tables S4 and S5, giving for each experiment the different parameters used, to guarantee reproducibility of the procedure.

Table 1 : How exactly is the importance parameter defined?

The sentence : “In the first generation, variables are weighted based on the chemical intuition from the solvothermal synthesis, as listed in Table 1” is not fully clear. If I understood the concept of the work, it is the intention to automate as much as possible the synthesis conditions, it is not clear what the authors mean by chemical intuition.

Page 6 : It is mentioned that the crystallinity was ranked on a scale of 1 to 10. It is not fully clear which metric the authors used to define the scale. Could a metric as introduced in http://dx.doi.org/10.1002/anie.202017153, be useful? Are the original XRD patterns obtained in reference 41, also taken up as reference?

As mentioned before the genetic algorithm of SycoFinder should be better introduced, as to ensure that readers understand how the second generation of experiments was found.

The authors used nitrogen adsorption isotherms to determine the surface areas. Are the data on this, fully reported in the main or supporting information?

The impact of the work could even be increased, if the authors introduce a paragraph/section on the computational/experimental effort necessary to use their methodology. In how far is the approach usable by other scientists in the field?

Figure 3 : Explain better the exact definition of the relative importance which is plotted in the pie chart.

The work of the authors allows to nicely detect which synthesis parameters are important to obtain crystalline materials with good yields. Is it possible, now that these conditions have been revealed, to comment on the chemical insights why some parameters are more or less important?

<u>Minor comments :</u>

In the abstract "to obtain the optimal synthesis of a MOF" to "to obtain the optimal synthesis conditions of a MOF"

Page 2 : Line 23 change "mechanochemical, microwave, and ultrasound" to "mechanochemical, microwave, and ultrasound based methodologies."

Page 2 : Line 23 change "In all these" to "In all these procedures"

Page 5 Lines 102-103. "and many were amorphous" change to "and many experiments yielded amorphous structures"

The authors might want to check whether they refer the figures in the right order, as they appear in the paper. For example Figure 4 is referred on Page 6, whereas the other figures – to the best of my knowledge – were not yet introduced.

Page 8 : Line 141 "we have summarized the results of this study. And in Figure" change to "we have summarized the results of this study and in Figure 3"

RESPONSE TO THE REVIEWERS

REVIEWER 1

The authors present a joint machine learning and experimental approach to optimize the synthesis conditions of Al-PMOF. This work is pretty original for the field of synthetic chemistry, as well as the use of algorithms towards synthetic chemists, the encouragement to communicate upon the failed experiments to improve knowledge and processes is also a relevant point for the community.

The idea and work developed in the manuscript are original and of true interest for the community of MOF chemists. Still, the work is sometimes explained too rapidly and some important data are not presented. Therefore, I encourage the authors to improve their manuscript prior to publication in Communications Chemistry.

Regarding the main manuscript, the following points are to be corrected/addressed in the text:

Reviewer Point P 1.1 — In the introduction, the discussion about the binding strength is confusing, as the concepts of kinetics and thermodynamics are mixed. This should be better explained.

Reply: This has been clarified in the introduction:

In all these procedures, the synthesis parameters play a major role in determining the crystal structure that forms as different conditions might stabilize different (meta)stable (intermediate) states.[1,2]

Reviewer Point P 1.2 — The authors mention only one synthesis condition reported to date (hydrothermal, high temperature). This should be balanced as there have been several reports where Al-PMOF could be obtained from alumina precursors in DMF/water mixtures at lower temperatures and in comparable solvents ratios presented in this manuscript. See the publications of G. Parsons on this matter (ex: https://doi.org/10.1016/j.matt.2019.11.005). I think it is worth mentioning these examples.

Reply: This has been taken into account in the revised version, and it now reads as follows:

Unlike HKUST-1, our knowledge of alternative synthesis conditions of Al-PMOF is limited. Some reports mention its synthesis using different reaction temperatures and aluminum precursors in a DMF:H_2O=1:3 [v/v] solvent mixture.[3,4] *However, the yield of these reactions is not reported.*

Reviewer Point P 1.3 — When discussing the low yield, the working up difficulties should be mentioned, especially to get rid of unreacted species in MOF types materials.

Reply: We wash the MOF with the organic solvent used for the synthesis. To highlight this point, the revised version now reads:

After synthesis, each sample was collected individually by centrifugation, washed with the organic solvent used for the reaction itself, followed by acetone, and finally dried overnight in a ventilated oven at 60°C. In some cases for which it seemed that some unreacted ligand was still present, DMF was also used. Working up the material with this type of solvent should help in the removal of unwanted products, in particular, the recrystallized porphyrin as it more soluble. Moreover, the crystallinity of the materials should always be assessed via PXRD measurements to address the purity of the structure and avoid the presence of any additional phase.

Reviewer Point P 1.4 — An important concern to me: from the manuscript and the SI it remains not clear to me why pure water was not included as a possible solvent for microwave tests? In fact, the authors test it with conventional heating and report reproducibility issues. They then test H_2O/DMF with conventional heating and report and optimized 80/20 ratio. It is very confusing why neither pure water nor H_2O/DMF mixture was tested in microwave sets of experiments? The paragraph 2, at page 4 does not provide any evidence on why these solvents are not tested. Please include these in the microwave test reactions and/or properly discuss why not.

Reply: This is a good point. The H_2O/DMF solvent mixture was studied in the microwave set of experiments (Figure 3, in the main text), and we have now included the microwave synthesis of Al-PMOF in pure water as well. We discussed both points further in detail in the "Experimental variables" section of the revised version of the manuscript:

The solvent composition was chosen to better solubilize all precursors (in particular porphyrin), which would help us achieve a high yield and crystallinity and minimize the amount of hazardous organic solvents used in the reactions. Preliminary results on solvothermal synthesis of Al-PMOF showed more reproducible yields and good crystallinity with a 80% H_2O:20% DMF solvent mixture (Table S2 and Figure S2). As solvents are deemed to be a critical factor in MOF synthesis as they can have a significant impact on the crystallization pathway and/or on the final product obtained,[5,6] we studied a total of five organic solvents with different boiling points (i.e., ethanol (EtOH), 1-propanol, dimethylformamide (DMF), dimethylacetamide (DMA) and dimethyl sulfoxide (DMSO)), which covered a wide range of temperatures from 75 °C to 190 °C. This would provide an additional degree of flexibility and parameters to study in our work.

as for the pure water microwave synthesis, we added the following in the "Method applicability and translatability" section of the revised version of the manuscript:

The microwave synthesis of Al-PMOF in pure water was also tested. The conditions used correspond to the best one obtained from generation 2 (i.e., G2S4). However, instead of a H_2O:EtOH solvent mixture, pure water was used (see Supporting Information for experimental details). The PXRD was crystalline and matched the calculated XRD from the CIF of Al-PMOF (Figure 1). However, the reaction yield was low and out of the three reactions performed, it only reached a maximum of 13% yield. These results suggest that the use of a co-solvent strongly helps in the synthesis of Al-PMOF with high yields.

Reviewer Point P 1.5 — Yields such a 90% in pure water seem very doubtful to me given the low solubility of the ligand in water and more importantly the fact that the reactions are performed in excess of porphyrin (therefore the max possible yield is below 100%).

Reply: The reviewer is correct. It may not have been clear in the manuscript that an approximation is used for the calculation of the yield as a high-throughput method. We divided the amount of MOF powder obtained by the amount of porphyrin ligand used in the synthesis. An accurate calculation of the yield would require thermogravimetric analysis (TGA) and elemental analysis (EA), which would be too time-consuming and laborious to perform for the 45 reactions done overall for generations 1 and 2.

We clarified this in the revised version:

An accurate calculation of the yield would require thermogravimetric analysis (TGA) and elemental analysis (EA). In this study, as a high-throughput approximation, we determined it by dividing the amount of powder obtained by the amount of porphyrin ligand used in the synthesis.

Reviewer Point P 1.6 — Page 5 “one-hot encoding for solvent type” what does this mean?

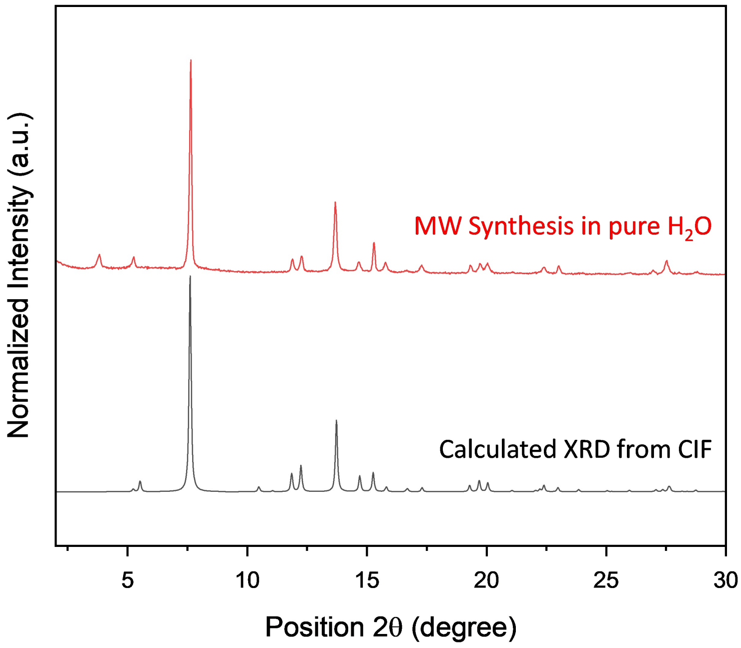

Figure 1: PXRD for the microwave synthesized Al-PMOF in pure water. This Figure was added in the revised version of the SI

Reply: We clarified this in the revised version:

In contrast to our previous work, where we treated solvents as a categorical variable described using a numeric array (so-called one-hot encoding in which the presence of a solvent is indicated with a 1 and absence with a 0), we describe solvents with their boiling points here. The boiling point is a critical factor when choosing solvents for a solvothermal synthesis.[5] *Using a chemically motivated descriptor for solvents can help the machine learning model better interpolate between different solvent types, leading to better predictions and interpretations.*

Reviewer Point P 1.7 — Regarding the solvents, why only the boiling point is considered? I think that much deeper discussion/work on solvent influence could be done. I guess the protic/aprotic type as well as the polarity are not less important matters.

Reply: The reviewer is correct and highlights the complexity of MOF synthesis. Although this would be highly interesting, it was not the focus of our work and with the limited amount of data, it would be uncertain to extrapolate any trends. We think that in order to be able to provide a more detailed analysis on the solvent influence (among other parameters on the outcome of the reaction), one would need to use multiple descriptors for the solvent and run several experiments to study these in particular. We added the following in the "Influence of the solvent" section:

Higher boiling point solvents (e.g., DMSO) show a much lower yield, while lower boiling point solvents (e.g., EtOH, 1-propanol) show a higher one (see Figure 3 in the main text). This suggests that pressure favors the crystallization of the MOF. However, this may not be the only descriptor which follows a linear trend with the results. Future work on the influence on the solvent in the outcome of the reaction could be performed for a better understanding of the crystallization process of Al-PMOF. For a more detailed analysis, one would need to use multiple descriptors for the solvent (e.g., polarity, proticity, etc.) and run several experiments to more accurately draw conclusive explanations.

Reviewer Point P 1.8 — The concept of "our chemical intuition" is not very clear. How would the intuition form one chemist to another impact on the whole study?

Reply: We clarified this in the revised version of the manuscript main text. With "chemical intuition", we refer to the set of unreported "tricks" that synthetic chemists use to identify the synthesis conditions of a material. In our work, we initiate the genetic algorithm with a set of 25 diverse experiments. The chemical intuition defines the weight of exploration for each variable in this initial set when we evaluate the similarity of synthesis conditions. We replace the word "importance" with "exploration weight" in Table 1 to further clarify this. In the revised manuscript, it reads as:

Based on the range of the variables given in Table 1, we used the SyCoFinder[7] to generate a set of 25 most diverse experiments, which covers the space of experimental variables as widely as possible (Table S4). To better initiate these experiments, we weighted the exploration of different synthesis variables with their importance quantified in our previous work using a machine learning model for the synthesis of HKUST-1.[8] Notably, in our previous work, we found that this chemical intuition (i.e., the importance of variables) is transferable to the synthesis of new materials. In addition, this chemical intuition matches our human chemical intuition based on our previous experiences with solvothermal synthesis. The weights for each variable are listed in Table 1.

Reviewer Point P 1.9 — In Figure 1, "crystal derived XRD pattern" and "predicted" are not the right formulations and could induce in error thinking that the Al-PMOF structure was solved from single crystal diffraction when it was solved from HR-PXRD data. The pattern is calculated from the CIF file and not predicted.

This figure should be changed to be much more informative. Please make a figure with much more patterns (at least one pattern per score from 1 to 8 for generation 1), showing the scores of different experiments so the crystallinity evolution along the scores become clearly visible. This way the phase purity/impurity will also be visible.

Reply: The authors agree with the reviewer and followed the recommendation by adding additional XRD patterns representing each crystallinity score attributed to samples of generation 1. The evolution of the crystallinity throughout generation 1 should now be more visible. The revised version of Figure 1 is shown below (Figure 2).

Reviewer Point P 1.10 — Please state clearly the best conditions obtained after the 2 generations run.

Reply: The best microwave conditions obtained have been detailed in the revised version which now reads as:

Al-PMOF can thus be efficiently synthesized in a microwave by inserting $AlCl_3 \cdot 6\,H_2O$ (0.099 mmol, 24 mg) and TCPP (0.051 mmol, 40 mg) in a solution of H_2O/EtOH (80%/20%) (2 mL). The vial is then sealed and inserted in the microwave for a 50 minutes reaction at 190 °C with 250 W of power.

Reviewer Point P 1.11 — Page 7: the authors say there was no need for a 3rd generation. I would still want them to discuss what would be the possibilities of a 3rd run? Would it be judicious/possible to introduce a new parameter from one generation to another based on the best performing conditions rather to optimize the same parameters?

Reply: Yes, we could add a third generation and seek, for example, for a higher surface area. However, given the data obtained, we would only expect marginal improvements as in just two generations, we were able to obtain a protocol which provides Al-PMOF with a high yield, good crystallinity and similar pore volume

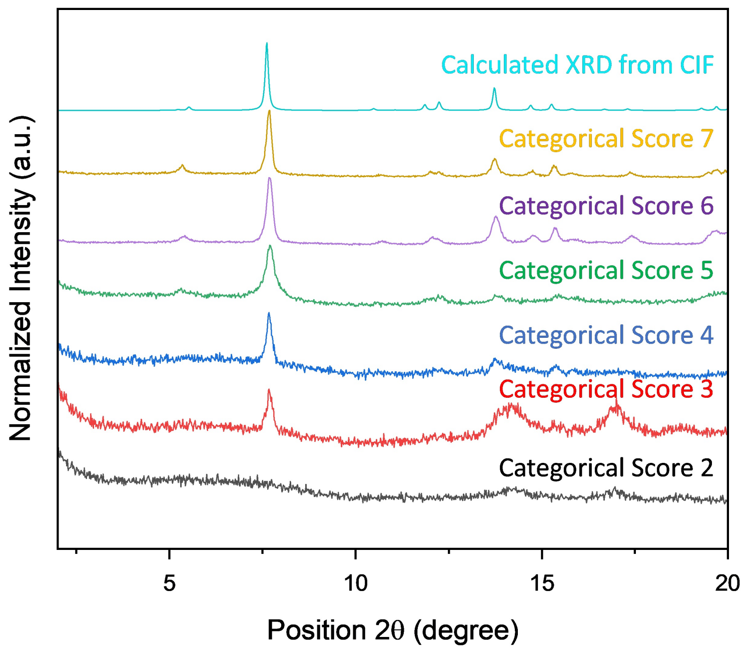

Figure 2: Revised version of Figure 1: PXRD of the best and worst samples produced from the first generation of experiments, with the calculated XRD from the CIF of Al-PMOF. The categorical score was given on a scale from 1 (worst) to 10 (best) in terms of crystallinity.

and CO_2 uptake compared to the previously reported one. Such process, may not be worth the time nor resources.

The revised version now reads:

This highlights the importance of the SyCoFinder in optimizing synthesis conditions. The methodology learns from the failed and partially successful experiments and discards conditions which do not yield the desired product. A failed or successful synthesis is judged on criteria defined by the ultimate goal of the study: if one is seeking good crystallinity, high yield or surface area (among other characteristics), a MOF that does not fulfill those requirements would be ranked worst and similar synthesis conditions would less likely be suggested in the following generations. This would lead us towards the completion of our goal in synthesizing a MOF with the characteristics of our interest. In our case, as the crystallinity and yield were sufficiently high and the surface area similar to what was previously obtained, there was no need for a 3rd generation of experiments, as we would only expect minor improvements.

Reviewer Point P 1.12 — Figure 2: should be more informative; provide the corresponding exact yield for each pattern.

Reply: The revised version of Figure 2 presents now the respective yields of the best and worst patterns obtained for generation 2 (Figure 3).

Reviewer Point P 1.13 — Page 8: hydrothermal instead of solvothermal

Reply: The manuscript has been changed accordingly.

Reviewer Point P 1.14 — The experimental data for the N_2 sorption isotherms should be provided.

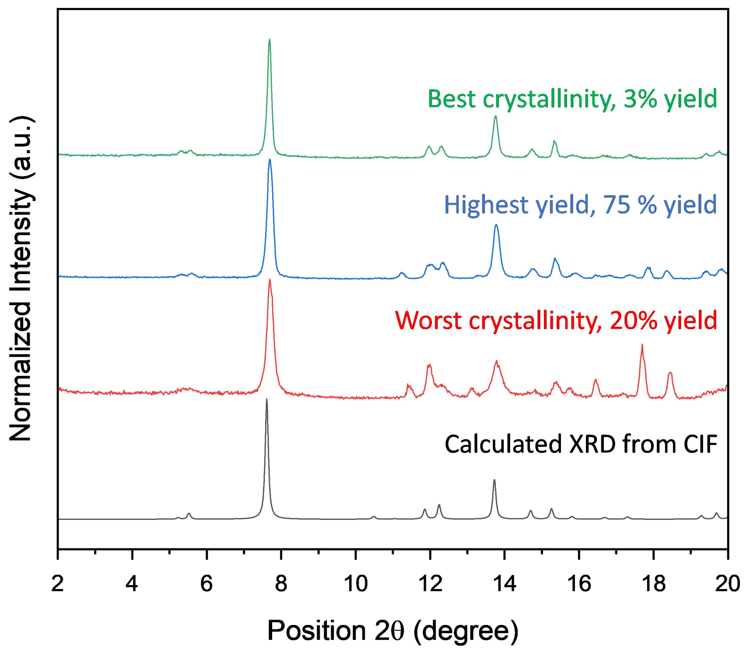

Figure 3: Revised version of Figure 2: PXRD of the best (highest crystallinity and yield) and worst crystallinity produced from the second generation of experiments, as well as the calculated XRD from the CIF of Al-PMOF.

Reply: All the characterization data (PXRD patterns, N_2 isotherms at 77K) is available on Zenodo (DOI: 10.5281/zenodo.7186602) and can be visualized through the following view developed with the visualizer library: `https://www.cheminfo.org/flavor/zenodo/index.html?id=&id=7186602`. As recommended by the reviewer, we have also added the N_2 adsorption isotherms in the SI (Figure 4, below). It now reads as follows:

The N_2 isotherms at 77 K for the most crystalline materials from generation 2: samples 4 and 15, are shown in Figure 4. The BET surface areas correspond to 1024 and 1226 $m^2 g^{-1}$, while the pore volumes are 0.687 and 0.736 $cm^3 g^{-1}$, respectively.

Reviewer Point P 1.15 — Reproducibility and large MOF synthesis: the word "scale" is missing (the MOF isn't large).

Reply: Changed accordingly.

Reviewer Point P 1.16 — On the matter of large-scale synthesis experimental data are lacking. Please provide quantitative data and results in terms of yield obtained for large scale, exact mas of material, the N_2 and CO_2 sorption experiments should be provided (in the SI).

Reply: The data has been added in the "Reproducibility and Large-Scale MOF Synthesis" section of the revised version of the SI, and it now reads as follows:

" For the large-scale Al-PMOF synthesis, the conditions that yielded the best results in generation 2 were used (i.e., G2S4). The procedure was as follows: a mixture of $AlCl_3 \cdot 6\,H_2O$ (0.099 mmol, 24 mg) and TCPP (0.051 mmol, 40 mg) was inserted in a solution of H_2O/EtOH (80%/20%) (2 mL). The vial was then sealed and inserted in the microwave for a 50 minutes reaction at 190 °C with 250 W of power.) We synthesized five sets of 8 reactions each. After the syntheses, each set was combined into a single vial and the PXRD patterns were measured as shown in Figure 5 a). Once the crystallinity of all 5 sets had been assessed,

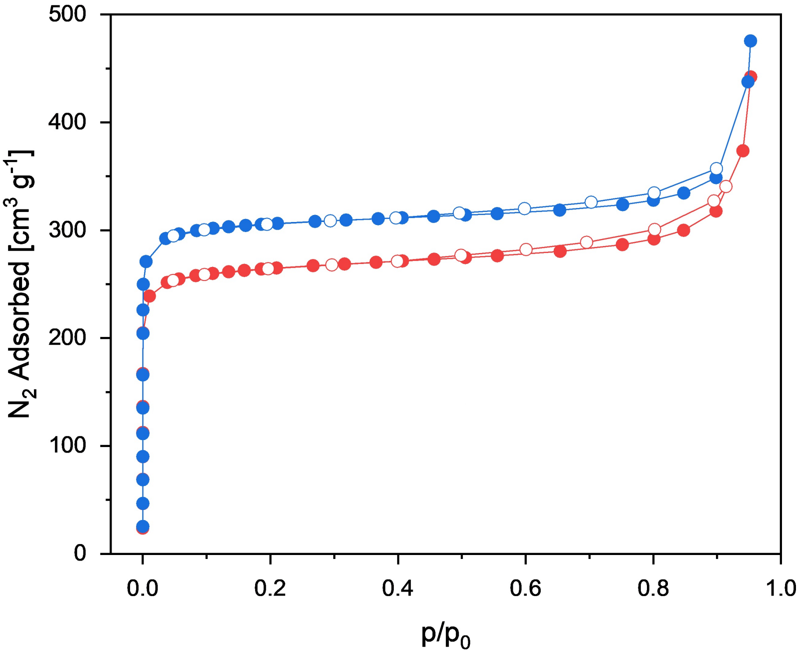

Figure 4: Single-component absorption (filled) and desorption (open) isotherms for N_2 collected on activated Al-PMOF at 77 K. The red and blue curves correspond to samples 4 and 15 of generation 2, respectively. This Figure was added to the revised version of the SI.

these were further combined into a larger batch whose yield was approximately 68%. The N_2 isotherm at 77 K of the combined batch was measured providing a BET surface area of 1264 cm^3 g^{-1} and pore volume of 0.628 cm^3 g^{-1} (Figure 5 b)). The CO_2 uptake was then measured after solvent exchange with acetone for 24 hours and activating the structure at 180 ° C under dynamic vacuum (Figure 5 c)). The uptake of 3.5 mmol/g at 1 bar is very close to the one reported in[9] (i.e., 4 mmol/g), which had been synthesized hydrothermally. This confirms that the synthesis of Al-PMOF in a microwave as we describe in this study provides similar pore structure as conventional heating and gives similar N_2 and CO_2 adsorption results."

Reviewer Point P 1.17 — Rather than multiplying the reactions, is it possible to perform the same reaction with the same yield and crystallinity in a larger reactor?

Reply: Due to the scope of our laboratory, which is mainly focused in MOF synthesis discovery rather than large-scale synthesis, this was not carried out in the study. There are, however, a variety of techniques for such purposes, such as spray drying, which could be used. Optimizing the conditions to synthesize Al-PMOF in a larger reactor could also be thought of.

Reviewer Point P 1.18 — The study presents very nice results stating that the synthesis could be performed in a less toxic solvent in a higher yield. For the sake of synthesis speed, the authors switched from hydrothermal to microwave synthesis. It would be of interest to check whether the microwave optimized conditions could be transposed to the conventional heating procedure. The time parameter will certainly be increased, but it is an important matter to discuss in terms of the adaptability of the optimized parameters to different kinds of synthesis equipment.

Reply: The authors agree with the reviewer and have carried out the reaction. The conditions used were based on the best conditions obtained with the microwave

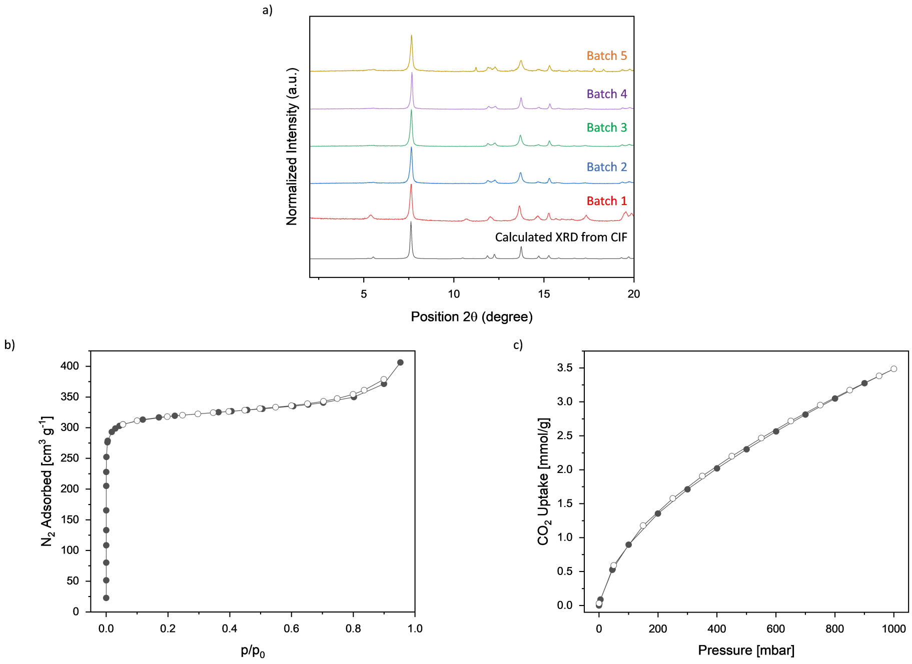

Figure 5: a) PXRD of all five batches of Al-PMOF synthesized in the robot; b) N_2 and c) CO_2 isotherms of the combined batches at 77 K and 298 K, respectively. Adsorption (filled) and desorption (empty) circles. This Figure was added to the revised version of the SI.

reactor. These, however, were adapted to a solvothermal synthesis in higher-volume (i.e., 23 mL) Teflon-lined autoclaves, and the reaction time was chosen to be the same as the one reported in the original hydrothermal synthesis (i.e., 16 hours). The revised version of the main text now reads:

The translatability of the optimized microwave conditions into a conventional heating procedure was also investigated. Al-PMOF was therefore solvothermally synthesized with the conditions that yielded the best-ranked material of generation 2 (sample 4 (i.e., G2S4), see Supporting Information for experimental details). The yields obtained ranged from 50 to 60 %. Similar to the microwave synthesis, also here the yield was calculated by dividing the amount of MOF powder obtained by the amount of TCPP ligand used in the synthesis. The PXRD pattern (Figure 6) confirms the crystallinity of the structure as it fully matches the calculated XRD from the CIF. The successful synthesis of Al-PMOF, along with the relatively high yield obtained, confirms the adaptability of the microwave-optimized parameters to different types of MOF synthesis equipment. This demonstrates the success of the SyCoFinder in optimizing synthetic conditions and the applicability of the method to a variety of materials and equipment.

and in the "Method Versatility" section of the SI:

The translatability of the optimized microwave conditions into a conventional heating procedure was also investigated. Figure 6 presents the PXRD pattern obtained. The time parameter was adapted and set the same as in the original hydrothermal synthesis (i.e., 16 hours). The amount of precursors was scaled-up to the 23 mL Teflon-lined bomb used: $AlCl_3 \cdot 6\,H_2O$ *(0.23 mmol, 55.2 mg) and TCPP (0.12 mmol, 92 mg) were mixed in 4.6 mL of a solvent mixture (80%* H_2O*:20% EtOH) and sealed in a 23 mL Teflon-lined bomb. The reaction was performed at 190 ° C for 16 hours with a heating and cooling rates of 2 and 0.2 °C/min, respectively. The PXRD pattern (Figure 6) confirms the crystallinity of the structure as it fully matches the calculated XRD from the CIF.*

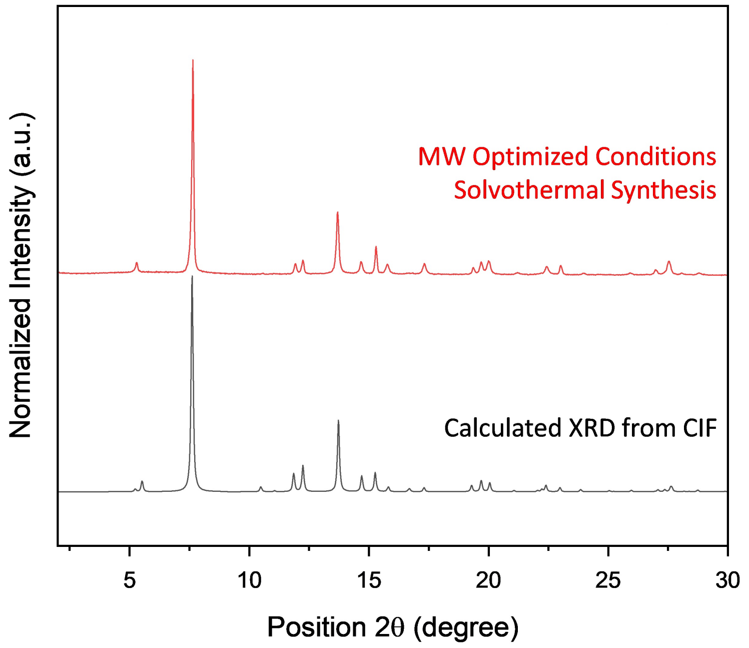

Figure 6: PXRD patterns obtained for the best-performing microwave conditions performed in a solvothermal reaction as well as the calculated XRD from the CIF of Al-PMOF. This Figure was added to the revised version of the SI.

Reviewer Point P 1.19 — In the first synthesis describe the figures in their order or change the order of figures.

Reply: Changed accordingly.

Reviewer Point P 1.20 — While performing microwave reactions with the assistance of robotic set up is very handy, I have a concern about the order of the reactions being tested. Indeed, 25 reactions are performed in the first run. This means that between the 1st and the last reactions, the mixtures are left at room temperature for very different times (over 10 hours). This could have an impact on the reaction outcome. Please consider this parameter and comment whether this is a variable that impacts the synthesis. For how long can the mixture be left standing without noticeable impact?

Reply: The reviewer makes a valid point. We assume the concern is related to the slightly acidic conditions that can be created due to the hydrolysis of the $AlCl_3$ precursor in water. We have therefore included the discussion of this point in the revised version of the main text, which now reads:

For the large-scale MOF synthesis, since the reactions run sequentially one after the other, the first reaction mixtures are left at room temperature in the mother solution for over very different times, whose longest could reach 16 hours. It is therefore important to assess the stability of Al-PMOF in slightly acidic conditions for an extended amount of time as the hydrolyzed aluminum salt makes the solution slightly acidic. In its original publication,[10] it is demonstrated that this structure is stable under acidic solutions (i.e., pH = 5). Moreover, Oliver T. Wilcox, et al.[11] have also reported Al-PMOF after loading it with different acids (hydrochloric acid (HCl) and formic acid) for 16 hours, confirming the remarkable stability of the MOF under acidic conditions. We are therefore confident that leaving the MOF in the mother solution overnight would not have a large effect on its crystallinity and pore structure. For other MOFs, however, this may be a factor that should be considered. In the

case of robotic synthesis, one possibility would be to automate a filtration and washing step of the sample after synthesis.

Reviewer Point P 1.21 — Page S3: “was dissolved” is not appropriate, a suspension would for given the low solubility.

Reply: Changed accordingly.

Reviewer Point P 1.22 — Please indicate the moles for each reactant rather than just the mass.

Reply: The manuscript has been changed accordingly.

Reviewer Point P 1.23 — Given the low scale of the reactions run, is it really possible to deduce the yield with a precision below 1% (for ex 3.5% in table S5).

Reply: The reviewer is correct. However, as replied in P 1.5, this only corresponds to an estimation of what the real yield would be.

REVIEWER 2

In their work, Domingues and coworkers report the use of a genetic algorithm to find better synthetic conditions for a promising MOF for CO2 capture, Al-PMOF. Various reaction conditions are varied through the genetic algorithm including temperature, reaction time, concentration, and solvent boiling point. It's a nice example and extension of the previous HKUST-1 work but applied to a different MOF with less synthetic data available. The insights provided on the effects from the different synthetic variables on the figures of merit of yield and crystallinity are interesting. It showcases the GA approach as a viable methodology to study synthesis conditions for MOFs on a larger scale.

Reviewer Point P 2.1 — Overall, the manuscript contains enough details except for how solvent is treated in the GA. The authors mention that one-hot encoding was used previously but now they describe the solvents with a continuous variable. It is unclear how that was done. They could have meant that it was strictly the boiling point of the solvent, but that also seems strange since they only include five different solvents each with its own boiling point, so it's not really continuous. This point needs to be made clearer. Once that is done, I think the manuscript is ready for publication.

Reply: We agree with the reviewer and we clarified this in the main text:

The ranges of these variables were based on our experience with the solvothermal synthesis of Al-PMOF and are detailed in Table 1. In contrast to our previous work, where we treated solvents as a categorical variable described using a numeric array (so-called one-hot encoding in which the presence of a solvent is indicated with a 1 and absence with a 0), we describe solvents with their boiling points here. The boiling point is a critical factor when choosing solvents for a solvothermal synthesis.[5] Using a chemically motivated descriptor for solvents can help the machine learning model better interpolate between different solvent types, leading to better predictions and interpretations. Finally, for the new synthesis conditions suggested by the genetic algorithm, we choose the solvent with the closest boiling point.

REVIEWER 3

The work of Smit et al. is a very interesting and original contribution, showing a methodology to systemically find the synthesis conditions for Metal Organic Frameworks that yield materials with high crystallinity and yield. The work builds further on their earlier contributions in the field. The methodology aims to systematically perform synthesis of materials in a directed way, in contrary to the trial and error approach that is commonly used. The work is an interesting contribution to the MOF field and might stimulate researchers to also publish their failed experiments. The work deserves to be published after some clarifications have been made as detailed below.

Reviewer Point P 3.1 — To improve the impact of the work for the broader scientific community, it is advised that the authors comment on the general applicability of the method, in how far it is usable for other researchers in the field. Furthermore, it is suggested that the authors motivate better why they used the particularly chosen MOF and elaborate on the general applicability to a broader set of MOFs and other synthesis protocols.

Reply: The authors agree with the reviewer's comment and have added the following to the "method applicability and translatability" section of the revised version of the main text:

MOF crystallization is a complex molecular process, and the synthesis recipes vary greatly from MOF to MOF. Therefore, it is important that our screening approach is versatile and easily adaptable to different synthesis optimization problems. The first step is to define the chemical space that we want to explore, which can be easily tuned according to our structure of interest, needs and the variables that one wants to optimize. Then, in contrast to the conventional "study one-factor-at-a-time", here, the SyCoFinder generates the most diverse set of syntheses conditions, which interestingly leads chemists to reaction conditions that probably would have never explored otherwise. Then, GAs and ML iterate the data towards a successful synthesis which provides us with the best target that fulfills our requirements (e.g., good crystallinity, high yield, high surface area, etc.). This is simply a quantified "intuition" developed by the ML model, which is similar to the intuition developed by experienced chemists in the lab. The advantage here is that the software used in this study is open access and available as a web application on the Materials Cloud.[7]

As for the choice of the MOF, we introduced the structure better and the interest of porphyrin-based MOFs in the following P 3.2.

Reviewer Point P 3.2 — It is advised to introduce the material under investigation "Al-PMOF" better, show the chemical structure of the building blocks and the structure of the finally obtained material. It is suggested to insert this clarification in the introduction, when the material is first mentioned.

Reply: The authors agree with the reviewer and have added the following to the introduction:

In this work, we applied the Synthetic Conditions Finder (SyCoFinder),[7] *which is the web-application based on the methodology developed by Moosavi et al.,*[8] *to find the optimal synthesis conditions for Al-PMOF ($Al_2(OH)_2$TCPP) [H_4TCPP = 4,4′,4″,4″-(porphyrin-5,10,15,20-tetrayl)tetrabenzoic acid], a porphyrin-based MOF first synthesized by Fateeva et al.*[10] *Publications of porphyrin-based MOFs have been exponentially increasing in the past 20 years*[12] *as this organic ligand has interesting characteristics and versatile functions, which makes it suitable for a wide range of applications. Thanks to their high visible-light absorption and energy transfer properties, porphyrins are very often used in solar cells, fluorescence imaging, and molecular probe applications.*[13] *The structure of Al-PMOF relies*

on one-dimensional chains of Al(III) running along the b-axis connected by the TCPP units through the carboxylate groups (Figure 7 a) and b)). The high surface area of this MOF and the stacks of the porphyrin ligand along the b-axis make this structure suitable for CO_2 capture in a wet environment, typical of flue gas from a coal-fired power station.[14]

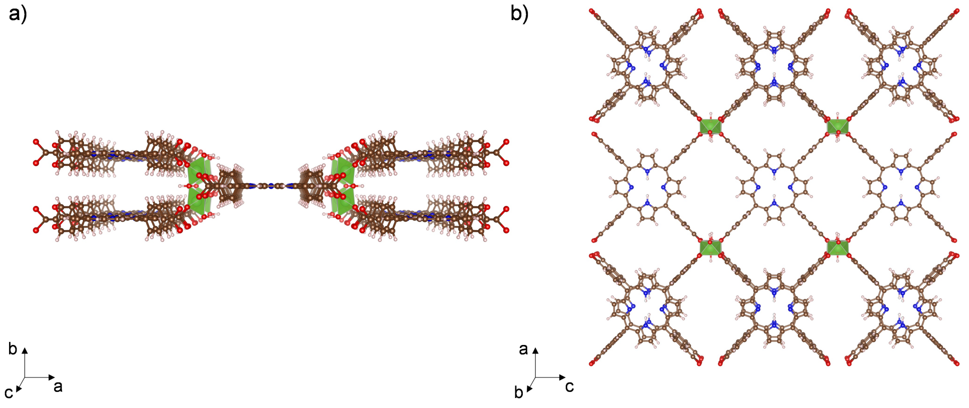

Figure 7: Ball-and-stick representation of Al-PMOF along the c and b axis, for Figures a) and b), respectively. Color code: C (brown), O (red), N (blue), H (light pink), Al (green). This Figure was added to the revised version of the main text.

Reviewer Point P 3.3 — The authors used a high-throughput microwave-based robotic platform to test various parameters of the synthesis conditions. On page 5, it is mentioned that the SyCoFinder was used to generate the 25 most diverse experiments, however this statements not fully clear based on the information given in the paper. The SyCoFinder methodology should be better introduced. It is advised to refer better to the tables S4 and S5, giving for each experiment the different parameters used, to guarantee reproducibility of the procedure.

Reply: We agree with the reviewer and now we have included a subsection to our method to introduce the SyCoFinder:

The synthesis condition finding procedure is adapted from our previous work.[8] *In this procedure, we initiate our experiments with a generation of the most diverse set of experiments identified using the farthest point sampling (i.e., MaxMin diversity). In this approach, to come up with N trials, we first add a trial chosen randomly. Then, for the other N-1 trials, we iteratively add the most dissimilar synthesis conditions to the set of previously selected trials, where we maximize the minimum distance to the currently selected trials. Here, the dissimilarity metric is the euclidean distance between two synthesis conditions weighted with an exploration factor that is listed in Table 1. Synthesis variables with higher weight are explored more.*

After this first generation, we use a genetic algorithm (GA), which is a global optimization algorithm, to explore the synthesis conditions space that we identified in Table 1 The GA uses genetic operations, including selection, crossover, and mutation, to generate new offspring from the previous generations. In this approach, two trials from the previous generation (parents) are selected, and their synthesis variables (genes) are combined using a crossover operation to generate a new synthesis trial (offspring). To include a chance to explore beyond the previous generations, some of the genes can mutate. The ratio between crossover and mutation balances the exploration vs. exploitation for the optimization. The synthesis trials with higher scores have a higher chance of being selected to transfer their genes to the next generation. As we use a ranking-based selection algorithm, the score function can be easily adapted to any target, e.g., crystallinity, yield, etc. The details of the genetic algorithm, including the crossover and mutation and the diverse set computations, are reported in our previous work.[8]

We also added a paragraph that better refers to all the different parameters used for each reaction of generations 1 and 2:

" Detailed synthesis conditions for each Al-PMOF reaction performed in this study can be found in the Supporting Information (Tables S4 and S5). The syntheses were carried out in a microwave synthesis reactor (Biotage, Uppsala, Sweden) which is connected to a high-throughput robotic platform (Chemspeed technologies, Füllinsdorf, Basel, Switzerland) (Figure S1.) The microwave is completely automated and executed with the Chemspeed autosuite software. All chemicals were purchased from commercial sources and used without further purification."

Reviewer Point P 3.4 — Table 1 : How exactly is the importance parameter defined?

Reply: Here we have not been very clear. In the revised manuscript, we explain what importance means, how it is quantified, and how it is put in use in the generation of the first synthesis trials using the farthest point sampling. Please, see our response to P 1.8 and P 3.3.

Reviewer Point P 3.5 — The sentence : "In the first generation, variables are weighted based on the chemical intuition from the solvothermal synthesis, as listed in Table 1" is not fully clear. If I understood the concept of the work, it is the intention to automate as much as possible the synthesis conditions, it is not clear what the authors mean by chemical intuition.

Reply: We clarified this in our response to P 1.8.

Reviewer Point P 3.6 — Page 6 : It is mentioned that the crystallinity was ranked on a scale of 1 to 10. It is not fully clear which metric the authors used to define the scale. Could a metric as introduced in http://dx.doi.org/10.1002/anie.202017153, be useful? Are the original XRD patterns obtained in reference 41, also taken up as reference?

Reply: We understand the reviewer's concern. We added the following to the "crystalline structure and yield" section of the main text:
Each PXRD pattern was analyzed individually and ranked qualitatively, depending on which pattern would match best the calculated XRD from the CIF of Al-PMOF. Evaluating the PXRDs by eye allows us to look at the spectrum as a whole rather than individual peaks and can give good insights into the crystallinity of the structure. Automatically ranking PXRD patterns using computer software could also be considered an objective measure that would allow for a more systematic analysis of the data obtained. However, it is highly complex to develop robust software which would take into account all experimental artifacts which could be misleading in some cases (e.g., amorphicity, unreacted ligand, etc.). Further work to assess the correspondence among PXRD patterns with a more accurate, systematic, and quantitative measure could be done. An example of how this could be implemented is thoroughly described in.[15]

Reviewer Point P 3.7 — As mentioned before the genetic algorithm of SycoFinder should be better introduced, as to ensure that readers understand how the second generation of experiments was found.

Reply: Agreed and revised, please see P 1.8 and P 3.3.

Reviewer Point P 3.8 — The authors used nitrogen adsorption isotherms to determine the surface areas. Are the data on this, fully reported in the main or supporting information?

Reply: As in the response to reviewer 1 (P 1.14), this data has been added in the revised version of the SI.

Reviewer Point P 3.9 — The impact of the work could even be increased, if the authors introduce a paragraph/section on the computational/experimental effort necessary to use their methodology. In how far is the approach usable by other scientists in the field?

Reply: We agree and have further discussed this point in the Method section of the revised version. Please, see P 3.1 and P 3.3.

Reviewer Point P 3.10 — Figure 3 : Explain better the exact definition of the relative importance which is plotted in the pie chart.

Reply: We added this in the revised manuscript:

We use SHAP (SHapley Additive explanations) values to quantify the importance of variables. SHAP values quantify how each variable influences the outcome of the machine learning model using a game theoretic approach.[16,17]

Reviewer Point P 3.11 — The work of the authors allows to nicely detect which synthesis parameters are important to obtain crystalline materials with good yields. Is it possible, now that these conditions have been revealed, to comment on the chemical insights why some parameters are more or less important?

Reply: We tried to address this in the discussion section. MOF synthesis is highly complex and it is hard to draw general conclusions from a limited amount of data (i.e., 45 reactions overall) in which a large number of variables were studied. As mentioned in P 1.7, future work should be performed to address the influence of each parameter on the crystallization pathway of Al-PMOF. For more conclusive explanations, additional experiments and multiple descriptors for each variable could be used to study each parameter individually and in a more detailed manner.

The relative importance of variables nicely points out that concentration and solvent are the most important factors for improving the crystallinity, while for the yield, solvent seems to play a major role (please, see the "Analysis of the experimental variables" section in the main text). Concentration can be positively correlated to the kinetics of the reaction. As these syntheses are all relatively fast (i.e., $<$ 1 hour), it seems important to have a higher concentration of the precursors to enhance the kinetics of the reaction, so that it may take place within the time available. Similarly, it seems that Al-PMOF is highly affected by the pressure inside the reaction vessel. Solvents with a lower boiling point could provide the optimal conditions for MOF crystallization (see "Influence of the solvent and concentration" sections in the main text).

Reviewer Point P 3.12 — In the abstract “to obtain the optimal synthesis of a MOF” to “to obtain the optimal synthesis conditions of a MOF”

Reply: Revised accordingly.

Reviewer Point P 3.13 — Page 2 : Line 23 change “mechanochemical, microwave, and ultrasound” to “mechanochemical, microwave, and ultrasound based methodologies.”

Reply: Revised accordingly.

Reviewer Point P 3.14 — Page 2 : Line 23 change “In all these” to “In all these procedures”

Reply: Revised accordingly.

Reviewer Point P 3.15 — Page 5 Lines 102-103. “and many were amorphous” change to “and many experiments yielded amorphous structures”

Reply: Revised accordingly.

Reviewer Point P 3.16 — The authors might want to check whether they refer the figures in the right order, as they appear in the paper. For example Figure 4 is referred on Page 6, whereas the other figures – to the best of my knowledge – were not yet introduced.

Reply: Revised accordingly.

Reviewer Point P 3.17 — Page 8 : Line 141 "we have summarized the results of this study. And in Figure" change to "we have summarized the results of this study and in Figure 3"

Reply: Revised accordingly.

REFERENCES

[1] Zhou, H.-C.; Long, J. R.; Yaghi, O. M. Introduction to metal–organic frameworks. 2012.

[2] Anderson, S. L.; Tiana, D.; Ireland, C. P.; Capano, G.; Fumanal, M.; Gładysiak, A.; Kampouri, S.; Rahmanudin, A.; Guijarro, N.; Sivula, K., et al. *Chemical science* **2020**, *11*, 4164–4170.

[3] Lee, D. T.; Jamir, J. D.; Peterson, G. W.; Parsons, G. N. *Matter* **2020**, *2*, 404–415.

[4] Zhao, Y.; Kornienko, N.; Liu, Z.; Zhu, C.; Asahina, S.; Kuo, T.-R.; Bao, W.; Xie, C.; Hexemer, A.; Terasaki, O., et al. *Journal of the American Chemical Society* **2015**, *137*, 2199–2202.

[5] Stock, N.; Biswas, S. *Chemical reviews* **2012**, *112*, 933–969.

[6] Van Vleet, M. J.; Weng, T.; Li, X.; Schmidt, J. *Chemical reviews* **2018**, *118*, 3681–3721.

[7] S.M. Moosavi, L. T.; Smit, B. Synthesis condition finder. 2019; https://www.materialscloud.org/work/tools/sycofinder.

[8] Moosavi, S. M.; Chidambaram, A.; Talirz, L.; Haranczyk, M.; Stylianou, K. C.; Smit, B. *Nature communications* **2019**, *10*, 1–7.

[9] Boyd, P. G.; Chidambaram, A.; García-Díez, E.; Ireland, C. P.; Daff, T. D.; Bounds, R.; Gładysiak, A.; Schouwink, P.; Moosavi, S. M.; Maroto-Valer, M. M., et al. *Nature* **2019**, *576*, 253–256.

[10] Fateeva, A.; Chater, P. A.; Ireland, C. P.; Tahir, A. A.; Khimyak, Y. Z.; Wiper, P. V.; Darwent, J. R.; Rosseinsky, M. J. *Angewandte Chemie International Edition* **2012**, *51*, 7440–7444.

[11] Wilcox, O. T.; Fateeva, A.; Katsoulidis, A. P.; Smith, M. W.; Stone, C. A.; Rosseinsky, M. J. *Chemical Communications* **2015**, *51*, 14989–14991.

[12] Zhang, X.; Wasson, M. C.; Shayan, M.; Berdichevsky, E. K.; Ricardo-Noordberg, J.; Singh, Z.; Papazyan, E. K.; Castro, A. J.; Marino, P.; Ajoyan, Z., et al. *Coordination Chemistry Reviews* **2021**, *429*, 213615.

[13] Chen, J.; Hu, C.-L.; Zhang, X.-H.; Li, B.-X.; Yang, B.-P.; Mao, J.-G. *Angewandte Chemie* **2020**, *132*, 5419–5422.

[14] Boyd, P. G. et al. *Nature* **2019**, *576*, 253–+.

[15] Borgmans, S.; Rogge, S. M.; De Vos, J. S.; Stevens, C. V.; Van Der Voort, P.; Van Speybroeck, V. *Angewandte Chemie International Edition* **2021**, *60*, 8913–8922.

[16] Lundberg, S. M.; Lee, S.-I. In *Advances in Neural Information Processing Systems 30*; Guyon, I., Luxburg, U. V., Bengio, S., Wallach, H., Fergus, R., Vishwanathan, S., Garnett, R., Eds.; Curran Associates, Inc., 2017; pp 4765–4774.

[17] Lundberg, S. M.; Erion, G.; Chen, H.; DeGrave, A.; Prutkin, J. M.; Nair, B.; Katz, R.; Himmelfarb, J.; Bansal, N.; Lee, S.-I. *Nature Machine Intelligence* **2020**, *2*, 2522–5839.

REVIEWERS' COMMENTS:

Reviewer #1 (Remarks to the Author):

After reviewing the revised manuscript and SI, along with the answers provided by the authors, I happily recommend to publish this work in Communications Chemistry

Reviewer #2 (Remarks to the Author):

Authors have adequately addressed my concerns.

Reviewer #3 (Remarks to the Author):

The authors have accounted for all my comments, the paper has substantially improved and can now be published as is.

Final reviewers' comments

• Reviewer 1: After reviewing the revised manuscript and SI, along with the answers provided by the authors, I happily recommend to publish this work in Communications Chemistry.

• Reviewer 2: Authors have adequately addressed my concerns.

• Reviewer 3: The authors have accounted for all my comments, the paper has substantially improved and can now be published as is.

Reply: The authors thank the reviewers.
